# Integrative Approach to Develop and Characterize Antibodies against the Cancer-Associated Antigen Sialyl Lewis A (CA 19-9)

**DOI:** 10.1021/jacsau.5c01596

**Published:** 2026-04-15

**Authors:** Anika Freitag, Sana K. Khilji, Ruslan Nedielkov, Shalini M Kumar, Michael Krummhaar, Janine Arndt, Gustavo M. S. G. Moreira, Jost Lühle, Felix Goerdeler, Carsten Kamphues, Maria A. Mroginski, Christian Roth, Peter H. Seeberger, Heiko M. Möller, Oren Moscovitz

**Affiliations:** † Department of Biomolecular Systems, Max Planck Institute of Colloids and Interfaces, 14476 Potsdam, Germany; ‡ Institute of Chemistry, 26583 University of Potsdam, 14476 Potsdam, Germany; § Institute of Chemistry and Biochemistry, Freie Universität Berlin, 14195 Berlin, Germany; ∥ Institute of Chemistry, 26524 Technische Universität Berlin, 10623 Berlin, Germany; ⊥ Chirurgisches Forschungslabor, Klinik für Allgemein- und Viszeralchirurgie, Charité -Universitätsmedizin, Campus Benjamin Franklin, 12203 Berlin, Germany; # Tacalyx GmbH, Magnusstr. 11, 12489 Berlin, Germany; ∇ Abteilung für Chirurgie in Weißensee, chirurgische Onkologie/Tumorchirurgie, Park-Klinik Weißensee, 13086 Berlin, Germany

**Keywords:** CA 19-9, sialyl Lewis A, pancreatic cancer, TACA, 1116-NS-19-9, glycan array, MD simulation, STD NMR

## Abstract

Sialyl Lewis A (sLeA), or the CA 19-9 marker, is the best validated and only FDA-approved serologic marker clinically used to monitor recurrence, progression, and therapy efficiency in pancreatic ductal adenocarcinoma (PDAC) patients, making it an attractive target for antibody development. Recent clinical trials have demonstrated satisfying safety profiles and unique expression in a range of malignancies that highlight CA 19-9 as an attractive TACA to target using a stand-alone drug or an adjuvant therapy. Hence, we set out to explore the use of synthetic sLeA in the development of additional monoclonal antibodies (mAbs) with enhanced sLeA recognition and improved efficacy. Two mAbs targeting sLeA were generated through mice immunization with synthetic sLeA glycoconjugates, synthetic glycan arrays, and hybridoma technology. We then compared the antigen-binding properties of the newly developed mAbs with those of the widely used mAb 1116-NS-19-9 and demonstrated improved affinity and specificity for native sLeA ectopically expressed in B16 melanoma cells, surpassing the performance of the established mAb 1116-NS-19-9. Of the two mAbs, GB11 was more promising. Therefore, to elucidate the structural origin of improved GB11’s antigen binding, we conducted high-resolution mapping of the molecular recognition patterns between sLeA and the different antibodies using X-ray crystallography and STD NMR. These analyses revealed subtle yet critical differences in the glycan engagement and identified key structural features underlying enhanced GB11’s recognition of sLeA. MD simulations further supported these observations, indicating distinct orientations of sLeA within the binding pockets of each mAb. Our results suggest improved recognition of the native sLeA antigen by the newly generated GB11 antibody, providing a detailed and high-resolution elucidation of the molecular interactions underlying this recognition. Our study provides a tool with improved theranostic properties against sLeA-overexpressing malignancies.

## Graphical abstract

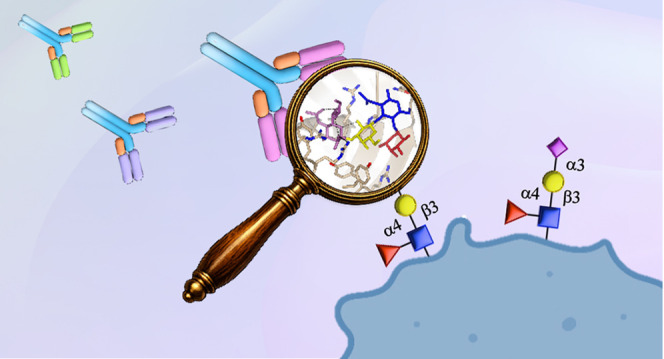

## Introduction

Glycosylation is the most abundant form of protein post-translational modification, taking part in every cellular process as part of the glycocalyx, including cell–cell adhesion, cell–matrix interaction, immune recognition, and membrane organization, among many others. It is not template-driven but mediated by coordinated functions of glycosyltransferases and glycosidases and takes place in the endoplasmic reticulum and Golgi compartments of eukaryotic cells. Glycosylation varies across cells and tissues and depends on the cell microenvironment, causing structural complexity and heterogeneity in glycans.^1–3^ Unsurprisingly, altered glycosylations are a hallmark of many diseases, including cancer. These aberrant tumor-associated carbohydrate antigens (TACAs) can, therefore, be used to distinguish cancer from healthy cells. TACAs are not only representative of the changes in neoplastic cell behavior but also play essential roles in the development, metastatic progression, and immune system evasion of cancer.^4–6^ Due to their abnormal structure or expression level on cancer cells, TACAs can be very attractive candidates for vaccine and antibody-targeted therapy.^7^ However, owing to their enormous diversity and structural complexity, obtaining sufficient homogeneous material of naturally derived glycans to immunize animals with and produce specific antibodies (Abs) again poses a considerable challenge.^8–10^ While total chemical synthesis can provide pure, well-defined glycans, it is laborious and time-consuming. The development of automated glycan assembly has accelerated and simplified the process significantly.^11^

Sialyl Lewis A (sLeA), also known as the CA 19-9 marker, is a tetrasaccharide composed of Neu5Ac­(α2–3)­Gal­(β1–3)­[Fucα(1–4)]­GlcNAc. Sialyl LeA is part of the human histo-blood system, known as the Lewis antigen system, which consists of the type I and type II Lewis antigens.^12^ H1, H2, Lewis A (LeA), LeB, LeX, and LeY share the same three monosaccharides, namely, N-acetylglucosamine (GlcNAc), galactose (Gal), and fucose (Fuc), differing only in their corresponding glycosidic bonds (type I: Gal­(β1–3)­GlcNAc; type II: Gal­(β1–4)­GlcNAc). Further addition of sialic acid (N-Acetylneuraminic acid (Neu5Ac)) to LeA and LeX leads to sLeA and sLeX, respectively.^12^

While it is found in embryonic tissue and is present at low levels in healthy tissue, sLeA synthesis is upregulated in specific epithelial cancers, including digestive tract, liver, breast, lung, pancreatic, and ovarian cancer, as well as noncancer diseases, such as diabetes mellitus, obstructive jaundice, and pancreatitis (which can predispose to pancreatic cancer).^13–16^ Moreover, it stands as the only US Food and Drug Administration (FDA)-approved biomarker for monitoring pancreatic cancer progression, which is generally known for its exceptionally low 5-year survival rate (9%) and poor prognosis (∼5 months) postdiagnosis.^17–20^

Sialyl LeA interacts with E- or P-selectins present on endothelial cells, thereby facilitating tumor vascularization during metastasis and allowing cancer cells to migrate to the metastatic site.^14^ In healthy tissue, disialyl Lewis A interacts with the immunosuppressive siglec-7 and -9 receptors on macrophages/monocytes and CD8^+^ T cells to help maintain the immunological homeostasis. However, elevated expression of sLeA abrogates this interaction, leading to chronic inflammatory stimuli that may support cancer progression.^21^ Furthermore, abnormal sLeA expression promotes inflammation by recruiting circulating lymphocytes to peripheral lymph nodes and cancerous tissue.^22^ Reciprocally, an inflammatory environment can enhance the expression of tumor-associated sialylated antigens such as sLeX/A by pro-inflammatory cytokines that regulate the expression of sialyltransferases.^23^

The Database of Anti-Glycan Reagents (DAGR) currently lists 43 Abs targeting sLeA,^24^ including the monoclonal antibodies (mAbs), murine 1116-NS-19-9 and human 5B1, that are most commonly used for clinical diagnosis and therapy of pancreatic cancer, respectively.^25^ The cytotoxic 5B1 (now MVT-5873 or BNT321) is a high-affinity human mAb that was generated from blood lymphocytes of a breast cancer patient immunized with synthetic sLeA conjugated to KLH,^26^ and is currently part of several ongoing or recently finished clinical trials.^27–31^ 1116-NS-19-9 is an IgG1 that was generated in mice in the early 1980s by immunizing with five different human colorectal carcinoma (CRC) cell lines.^31–33^ While others have previously employed methods to improve upon the binding and characterization of antiglycan Abs, including 1116-NS-19-9,^34,35^ the question of how the carbohydrate homogeneity and mode of presentation affect the finally recognized carbohydrate epitope and affinity of carbohydrate-specific antibodies during the immunization process is still not fully understood. To evaluate how the “natural” immunization caused by aberrantly glycosylated cancer cells (such as in the case of 1116-NS-19-9) compares to the immunization with a well-defined carbohydrate antigen, we used a construct of synthetic sLeA conjugated to CRM_197_ (sLeA- CRM_197_) to immunize mice and isolated two murine antibodies, namely, GB11 and HA8, which we characterized and compared to the murine 1116-NS-19-9.

## Results

### New Antibodies Share a High Sequence Similarity

Following the immunization with synthetic sLeA conjugated to CRM_197_ (Figure S1), we obtained two novel antibodies, GB11 and HA8, using hybridoma technology. RNA isolation, followed by RT-PCR, was used to obtain DNA to determine the sequences of both mAbs. The light chains of GB11 and HA8 were identified as the κ-class (Figure S2). Comparison of the variable regions of heavy and light chain sequences with those of 1116-NS-19-9 (PDB ID: 6XTG) revealed remarkable identities of the variable regions: 97% for GB11 and 96% for HA8 in the variable region of light chains and 91% for GB11 and 89% for HA8 in the heavy chain sequences (Figure 1a–b). The sole differences in the binding regions are identified in the complementarity-determining region 2 (CDR2) in the heavy chains for both GB11 and HA8. Instead of the charged Lys and a polar Asn, GB11 features the hydrophobic Tyr and Ile at positions 54 and 56 (sequential numbering of amino acids), respectively. Likewise, HA8 displays a more hydrophobic CDR2, with Trp and Val instead of Lys and Gly at positions 54 and 55, respectively. The “conserved region” of GB11 (PDB ID: 9I9H) reveals several mutations in the CH1 and CL regions, leading to a reduced sequence identity of the heavy chain to 78% and of the light chain to 79% compared to 1116-NS-19-9 (Figure S3).

**Figure 1:**
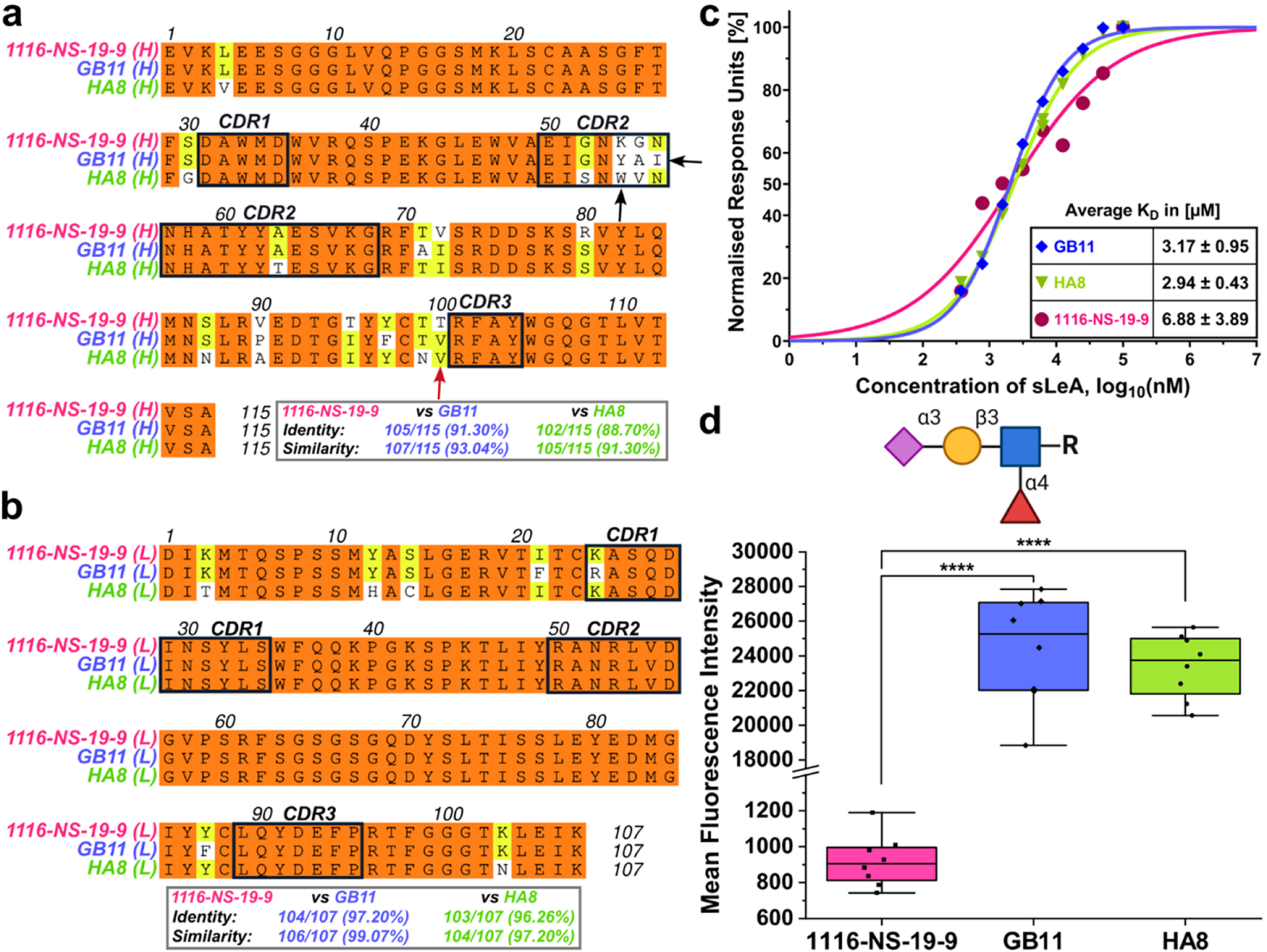
Enhanced binding of GB11 and HA8 to synthetic sLeA despite sequence similarity to 1116-NS-19-9. (a–b) Amino acid sequence alignment of heavy (H) and light (L) chain variable regions of 1116-NS-19-9, and the novel mAbs GB11 and HA8. Complementarity-determining regions (CDRs) are indicated by black boxes. Amino acid variations are indicated in white, with arrows highlighting potentially significant mutations. The red arrow signifies one of seven affinity-enhancing key mutations previously introduced by Borenstein–Katz et al.^25^ Identity and similarity scores relative to 1116-NS-19-9 are shown below. (c) SPR analysis showing binding of synthetic sLeA to captured mAbs 1116-NS-19-9 (magenta), GB11 (blue), and HA8 (green). One representative run per mAb is shown. Response units (RU) were normalized and plotted against the logarithmic sLeA concentration. Data were fitted using a variable-slope, dose–response model in GraphPad Prism (v10.4.2). *K*_D_ averages across three replicates are listed in the inset table. (d) Mean fluorescence intensity (MFI) of 1116-NS-19-9 (magenta), GB11 (blue), and HA8 (green) binding to synthetic sLeA, immobilized on a glycan array slide. Each mAb was tested in four independent binding assays. Data are presented as box plots; statistical analysis employed one-way ANOVA (**** *P* ≤ 0.0001). The glycan structure of sialyl Lewis A is depicted in “Symbol Nomenclature for Glycans” (SNFG) notation above (created with BioRender.com).

All three mAbs demonstrate comparable behavior in a thermal stability assay (Figure S4). HA8, GB11, and 1116-NS-19-9 all display relatively high melting points. HA8 has a melting point of 88 °C, while GB11′s melting point is 90 °C. Interestingly, 1116-NS-19-9 showed two melting points, with T1 at 79 °C and T2 at 91 °C. A shoulder in a similar temperature range was also observed for GB11 and HA8 (Figure S4); whether this corresponds to a real second melting point and, thereby, implies a biphasic behavior could not be determined conclusively.

### GB11 and HA8 Recognize Synthetic sLeA Exhibiting Similar Binding Modes

To evaluate the binding affinities of the generated antibodies against the synthetic immunization target, we employed both surface plasmon resonance (SPR) and isothermal titration calorimetry (ITC). Both methods independently determined dissociation constants (*K*_D_) in the micromolar (μM) range, consistent with typical affinities observed for antiglycan Abs.^36^

SPR analysis conducted in triplicate yielded *K*_D_ values ranging from 2.22 to 4.12 μM for GB11, 2.51–3.37 μM for HA8, and 2.99-10.77 μM for 1116-NS-19-9 (Figure 1c, Figures S6–S7). GB11 and HA8 thus exhibited slightly higher affinities than 1116-NS-19-9 (Figure 1c). Consistent with these findings, ITC measurements produced comparable *K*_D_ values, with mean dissociation constants of 1.66 ± 0.52 μM for GB11 and 1.67 ± 0.32 μM for HA8. The number of binding sites (N) determined with ITC was approximately 2 for both mAbs (Table 1), in line with a bivalent interaction in which each antigen-binding fragment (Fab) engages with sLeA independently. Moreover, the Gibbs free energies of binding (Δ*G*) were negative for both GB11 (−33.2 ± 0.8 kJ·mol^–1^) and HA8 (−33.1 ± 0.6 kJ·mol^–1^), indicating spontaneous and thermodynamically favorable interactions. The binding process was predominantly enthalpy-driven, with Δ*H* values of −22.7 ± 2.6 kJ·mol^–1^ for GB11 and −18.3 ± 0.5 kJ·mol^–1^ for HA8, accompanied by modestly unfavorable entropic contributions (*T*Δ*S* = −10.6 ± 3.4 kJ·mol^–1^ for GB11 and −14.7 ± 0.9 kJ·mol^–1^ for HA8) (Table 1). The similarity of these thermodynamic parameters suggests that both mAbs recognize sLeA through closely related binding modes. Representative titration curves and corresponding enthalpy fits are provided in Figure S5a–g.

**Table 1:** Affinity Measurements of GB11 and HA8, Obtained via ITC

**mAb**	***K***_**D**_ **[μM]**	**binding sites (N)**	**Δ*H*** [ kJ·mol^–1^ **]**	**Δ*G*** [kJ·mol^–1^ **]**	***T*Δ*S*** [kJ·mol^–1^ **]**
**GB**11 (1)	1.57	2.1	–23.9	–33.1	–9.3
**GB**11 (2)	0.82	1.8	–17.7	–34.7	–17.1
**GB**11 (3)	2.60	2.2	–26.4	–31.9	–5.5
**HA**8 (1)	1.04	1.8	–18	–34.2	–16.1
**HA**8 (2)	2.01	2.1	–19.4	–32.5	–13.1
**HA**8 (3)	1.97	2.	–17.6	–32.6	–15.0
**average GB11**	1.66 ± 0.52	2.0 ± 0.1	–22.7 ± 2.6	–33.2 ± 0.8	–10.6 ± 3.4
**average HA8**	1.67 ± 0.32	2.0 ± 0.1	–18.3 ± 0.5	–33.1 ± 0.6	–14.7 ± 0.9

To elucidate the antigen-binding specificity of the mAbs, we used synthetic glycan arrays. All three mAbs, GB11, HA8, and 1116-NS-19-9, bound exclusively to sLeA, with no detectable interaction with structurally related glycans, including those of other sialylated Lewis family members (Table S1, Figure S8a, S8e), confirming their high epitope specificity (Figure 1d, and S8b–d). Moreover, GB11 and HA8 exhibited significantly higher mean fluorescence intensities (MFI) than 1116-NS-19-9, suggesting enhanced binding to the immobilized, synthetic target antigen sLeA (Figure 1d). Specifically, HA8 demonstrated a 26.6-fold increase (mean MFI: 24,428.5 ± 3190.1) and GB11 a 25.5-fold increase (mean MFI: 23,413.9 ± 1867.5), relative to 1116-NS-19-9 (mean MFI: 916.6 ± 142.6).

### GB11 Demonstrates Better Binding to Native sLeA Compared to Other mAbs

GB11, HA8, and 1116-NS-19-9 bound native sLeA on patient-derived carcinoma tissue of pancreatic ductal adenocarcinoma (PDAC) and gastric adenocarcinoma (GAC) with no apparent differences and minimal binding to cancer-free pancreatic and gastric mucosa tissue (Figure 2a–b).

**Figure 2:**
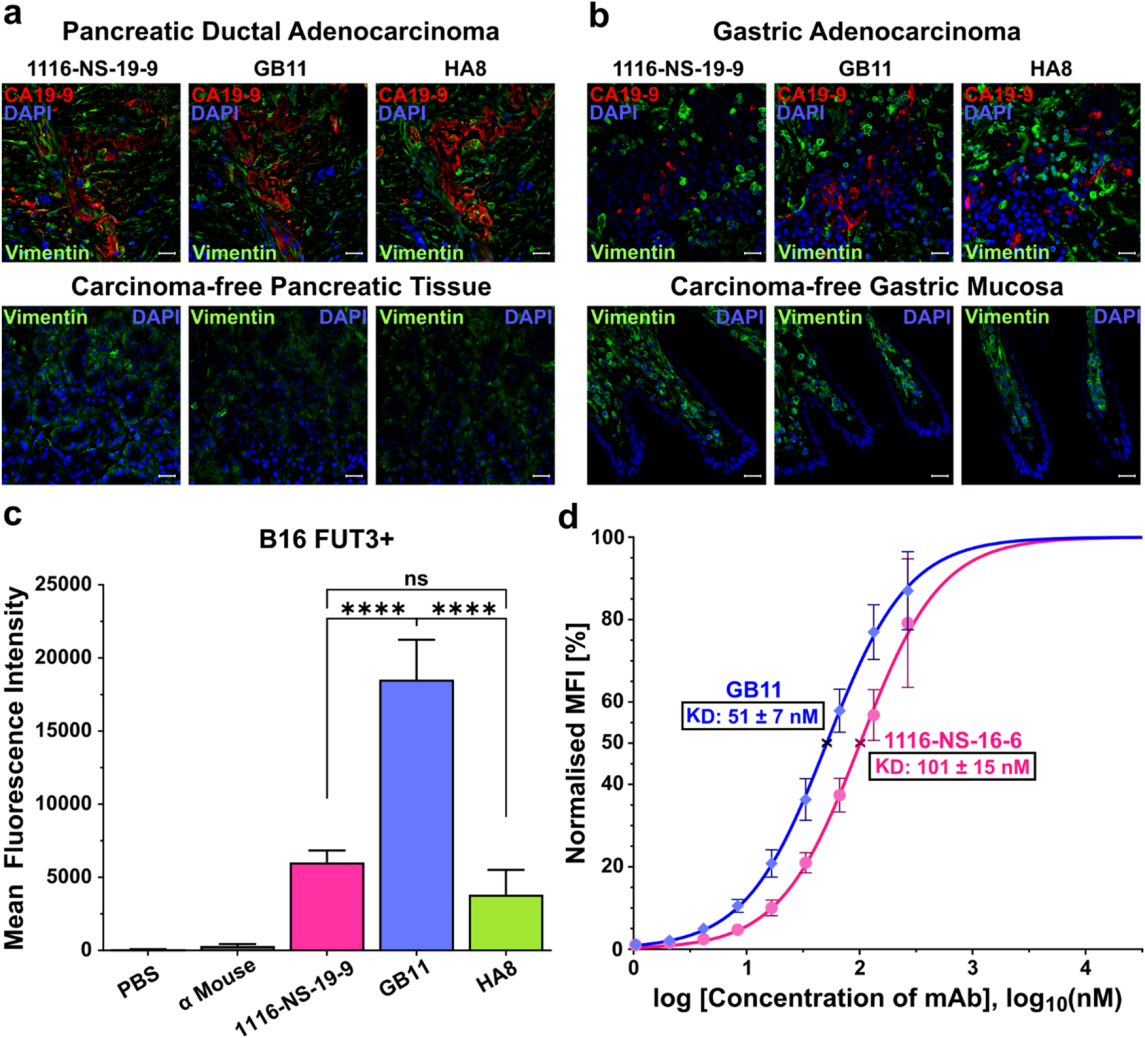
GB11 exhibits significantly stronger binding affinity to native sLeA. (a–b) Immunohistochemistry (IHC) revealed binding of α-sLeA mAbs as indicated with sLeA (red), DAPI-stained cell nuclei (blue), and Vimentin (green). The scale bar denotes 20 μm. (a) Upper: IHC of pancreatic ductal adenocarcinoma; lower: carcinoma-free pancreatic tissue. (b) Upper: IHC of gastric adenocarcinoma; bottom: carcinoma-free gastric mucosa. (c–d) Flow cytometry analysis on sLeA-expressing B16-FUT3+ mouse melanoma cell line reveals (c) MFI with SEM of 1116-NS-19-9 (magenta), GB11 (blue), and HA8 (green), with five independent assays per sample. Statistical analysis utilized one-way ANOVA (ns = not significant, ****P ≤ 0.0001). Additionally, a titration binding assay (d) for GB11 (blue) and 1116-NS-19-9 (magenta) was conducted, with MFI normalized relative to the highest measured MFI of each mAb. The dots represent the normalized MFI with its corresponding SEM. The fitted results illustrate the normalized MFI against an increasing concentration of each mAb over a logarithmic scale, with the *K*_D_ values marked with **x** on the fitted curve.

To further evaluate the binding of GB11, HA8, and 1116-NS-19-9 to the native epitope, flow cytometry measurements were conducted using the genetically engineered B16-FUT3+ cell line, which expresses the human fucosyltransferase III (FUT3), resulting in stable sLeA expression on the cell surface.^37^ The parental B16 cell line, which lacks sLeA expression, served as a negative control.

All three mAbs bound B16-FUT3+ cells (Figure 2c) but not the parental B16 line (Figure S9a), confirming their specificity for sLeA. Among them, GB11 turned out to be the best binder. It demonstrated the strongest signal in flow cytometry, with a significant 3-fold increase in MFI compared to 1116-NS-19-9 (Figure 2c), supporting the previous data for binding to a synthetic glycan. Interestingly, despite HA8 showing a similar binding profile to GB11 for synthetic sLeA, its cellular binding to native sLeA was weaker. GB11 and 1116-NS-19-9 bound 4.8-fold and 1.6-fold more strongly, respectively. Given GB11′s superior performance, especially for targeting native sLeA, GB11 and 1116-NS-19-9 were selected for further evaluation in a flow cytometry-based titration binding assay using B16-FUT3+ cells. This assay yielded apparent *K*_D_ values of 51 ± 7 nM for GB11 and 101 ± 15 nM for 1116-NS-19-9 (Figure 2d, Figure S9b).

### Preorganization of GB11′s Antigen-Binding Site Might Lead to Higher sLeA Affinity

Given the relatively minor sequence differences of GB11 and 1116-NS-19-9, yet the significantly higher binding properties of GB11 to synthetic and native sLeA, we sought to better understand their structural divergence by using X-ray crystallography. Structures of GB11 were successfully determined in both apo (PDB ID: 9I6Q) and antigen-bound (PDB ID: 9I9H) forms (see Table S2), while attempts to obtain crystals for HA8 were unsuccessful.

The GB11 Fab overlaps with 1116-NS-19-9 with RMSD values of 0.44 and 0.47 Å for the native and the ligand-bound structures, respectively. The sLeA antigen binds within a groove formed by all CDRs except for CDRL1 (Figure 3a). Despite the clearly stronger binding to sLeA by GB11 compared to that of 1116-NS-19-9, the 3D structures of both complexes are remarkably similar. The sLeA antigen adopts the same low-energy conformation in both structures while interacting with the same amino acids (See Table S3). For GB11, this interaction consists of a hydrogen bond (H-bond) network with Trp33, Asn53, Arg101 of the heavy chain, and Tyr91 and Arg96 of the light chain, along with a single salt bridge between Arg50 of the light chain and the carboxylate of Neu5Ac (Figure 3a).

**Figure 3:**
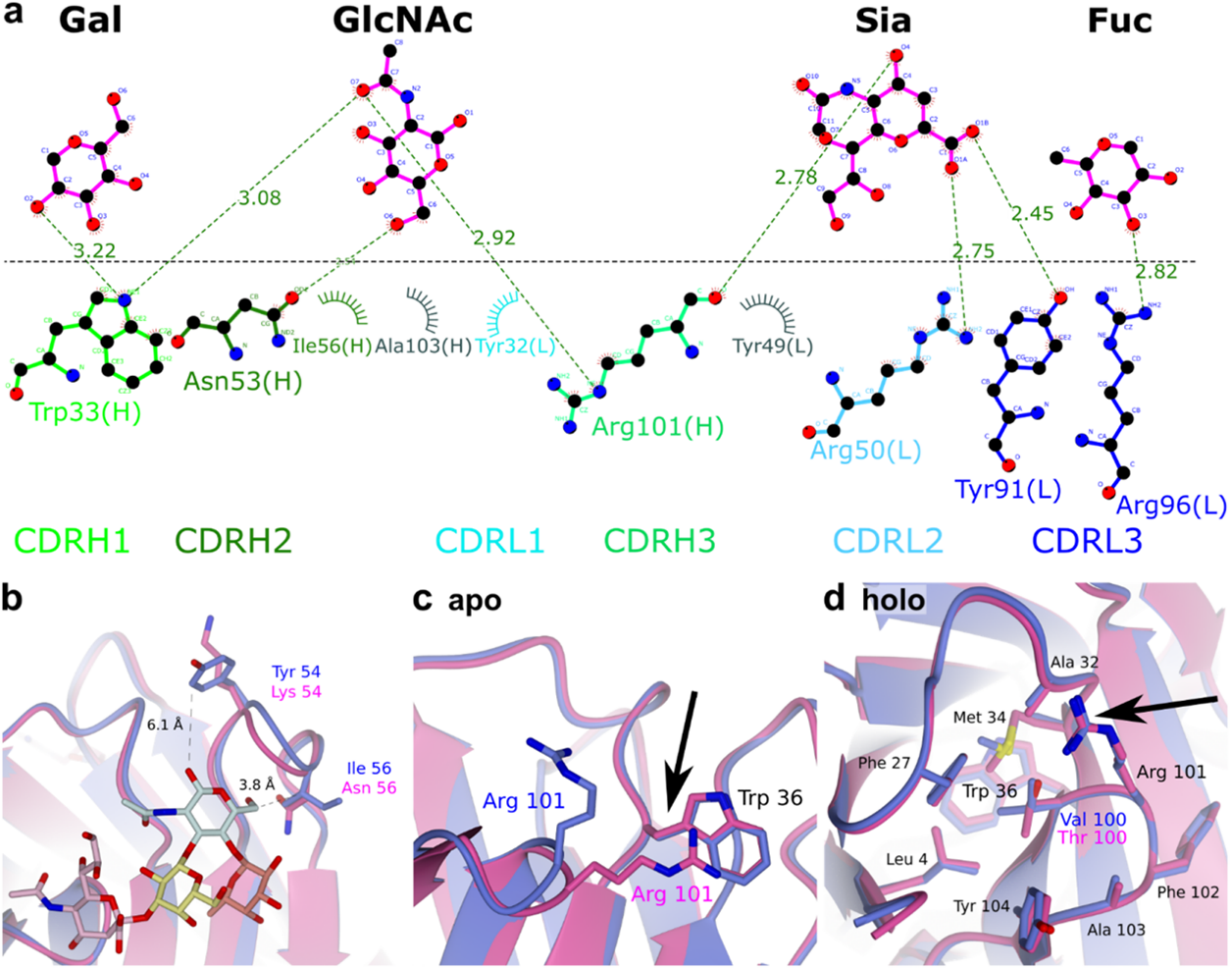
GB11 possesses an open rotamer configuration,unlike 1116-NS-19-9, which requires conformational change upon sLeA binding. (a) Interaction network between amino acid residues of GB11 and sLeA. (b) Overlay of the holo structure of GB11 (PDB ID: 9I9H; blue) and 1116-NS-19-9 (PDB ID: 6XTG; magenta), displaying side chains of mutations within the CDR of GB11 and their interatomic distances to the antigen sLeA. (c–d) Arg101 of 1116-NS-19-9 must undergo a conformational change upon binding, whereas GB11 is already in the binding conformation. (c) Overlay between the apo-structure of GB11 (PDB ID: 9I6Q; blue) and 1116-NS-19-9 (PDB ID: 6XUD; magenta). Arg101 in GB11 already adopts an open conformation, whereas in 1116-NS-19-9, it interacts with the nearby Trp36. (d) In the holo-structures, Arg101 of GB11 and 1116-NS-19-9 are in the same conformation, as indicated by the black arrow, within the hydrophobic pocket created by Val100 for GB11 and Thr100 for 1116-NS-19-9, as well as Leu4, Phe27, Ala32, Met34, Phe102, Ala103, and Tyr104.

Among the differing amino acids within the CDRH1, Tyr54 of GB11 appears to be positioned too far from the antigen to participate, with a distance of 6.1 Å. Conversely, Ile56 is located at a distance of 3.8 Å from the C6 of the GlcNAc, suggesting a potential involvement in a hydrophobic interaction (Figure 3b). The corresponding Asn56 (Asn53 Kabat numbering scheme^25^) in 1116-NS-19-9 creates a slightly more hydrophilic environment, but with a distance of 3.9 Å for the NH_2_ group and the geometry, it is unlikely to be involved in a direct H-bond.

However, the Thr100Val substitution within the variable heavy chain may play a more significant role in the binding to sLeA. This is supported by the fact that the Arg101 residue within the same loop forms a hydrogen bond with both GlcNAc and the Neu5Ac. The hydrophobic pocket, which the side chain of residue 100 must enter for the binding, may be stabilized by the higher hydrophobicity of Val (in GB11) over Thr (in 1116-NS-19-9). Indeed, while the 1116-NS-19-9 antibody’s apo-structure is reported to form a cation-π interaction between Trp36­(H) and Arg101­(H) (Trp33 and Arg95 Kabat numbering scheme^25^), and possibly also an H-bond between Glu50­(H) and Arg101­(H), these interactions appear to be absent in our GB11 antibody, with the Arg101 already displaying the same rotamer configuration as in the holo structure of 1116-NS-19-9 (Figure 3c–d). Thus, the antigen-binding site in GB11 is already preorganized to bind the antigen, while 1116-NS-19-9 needs to change its conformation.

### Molecular Dynamics Simulation Supports Better sLeA Binding Affinity of GB11

To gain deeper insight into carbohydrate recognition and link the high-resolution yet static details of the X-ray structure with the dynamic situation in solution, as reflected by our STD NMR studies (see below), we performed extensive MD simulations. An optimized workflow was applied to protein–ligand complexes to maximize data quality, comprising energy minimization, redocking, and thermal equilibration prior to a 200 ns MD production run. For comparison, the apo forms of both antibodies were simulated under identical conditions. To distinguish transient from stabilizing sLeA interactions, the final 100 ns of three 200 ns MD replicates per antibody were analyzed using root-mean-square deviation (RMSD), root-mean-square fluctuation (RMSF), radius of gyration (*R*_g_), molecular mechanics Poisson–Boltzmann surface area (MM-PBSA) binding energies, solvent-accessible-surface area (SASA), and interaction energy analyses.

RMSD analyses show convergence of 1116-NS-19-9 within the final 50 ns (RMSD < 2 Å) and GB11 within the last 20 ns (RMSD < 1.5–2 Å across replicates), while sLeA remains highly stable in both complexes (RMSD < 1 Å; Figure S11a–d).

Residue mobility was evaluated by RMSF, focusing on binding-site residues in apo and holo forms. sLeA binding reduced RMSF values in GB11 by up to 1 Å (Figure S12b), indicating increased rigidity, whereas no comparable reduction was observed for 1116-NS-19-9 (Figure S12a), suggesting a more stable binding interface in GB11.

Structural compactness was assessed using the radius of gyration (*R*_g_).^38^ Both antibodies remained highly compact in apo and holo forms, with R_g_ deviations <1 Å from the starting structures, confirming complex stability throughout the simulations (Figure S13).

MD-based MM-PBSA analysis^39,40^ of the final 10 ns indicates stronger sLeA binding to GB11, with average binding energies of −5.2 kcal/mol for GB11 versus −3.5 kcal/mol for 1116-NS-19-9. Consistently, average antibody sLeA interaction energies were −68.4 and −65.3 kcal/mol, respectively (ΔΔ*H* ∼3.1 kcal/mol). The lowest-energy frames further emphasized this trend (−87.6 vs −81.7 kcal/mol), corresponding to a ∼6 kcal/mol stronger interaction for GB11 (Figures S14, S15).

These trends are rationalized by residue-level analysis of binding modes and interactions within the Ab binding sites.^41,42^ Persistent hydrogen bonds observed across all simulation replicates are summarized in Figure 4a with H- bond occupancies reported as percentages of simulation time. In 1116-NS-19-9, stable H-bonds are dominated by interactions with Neu5Ac via Arg101­(H), Arg50­(L), and Tyr91­(L) (∼99%), with additional contacts to Gal and GlcNAc, while Fuc does not form persistent H-bonds. In contrast, GB11 forms stable H-bonds with all four sLeA residues, including persistent Fuc interactions with Glu50­(H), Tyr91­(L), and Arg96­(L) (>90%), alongside Neu5Ac, Gal, and GlcNAc contacts, indicating a more extensive and stable hydrogen-bonding network. Furthermore, analysis of the MD simulations shows that Trp33­(H) in 1116-NS-19-9 forms a transient CH−π contact with sLeA GlcNAc (∼22% of the final 100 ns; Figure S16). In contrast, GB11 exhibits multiple, more persistent, Trp33­(H)-mediated CH−π interactions. Arpeggio analysis of low-energy conformations revealed a single contact in 1116-NS-19-9 (Fuc C6; 3.7 Å, 34.1°), whereas GB11 forms additional face-on contacts with GlcNAc (BGL C4; 4.2 Å, 20.6°) and Fuc (C2; 3.8 Å, 25.9°), along with edge-to-face packing (BGL C4-CD2; 4.7 Å), supporting stronger anchoring and restricted sLeA mobility.

**Figure 4:**
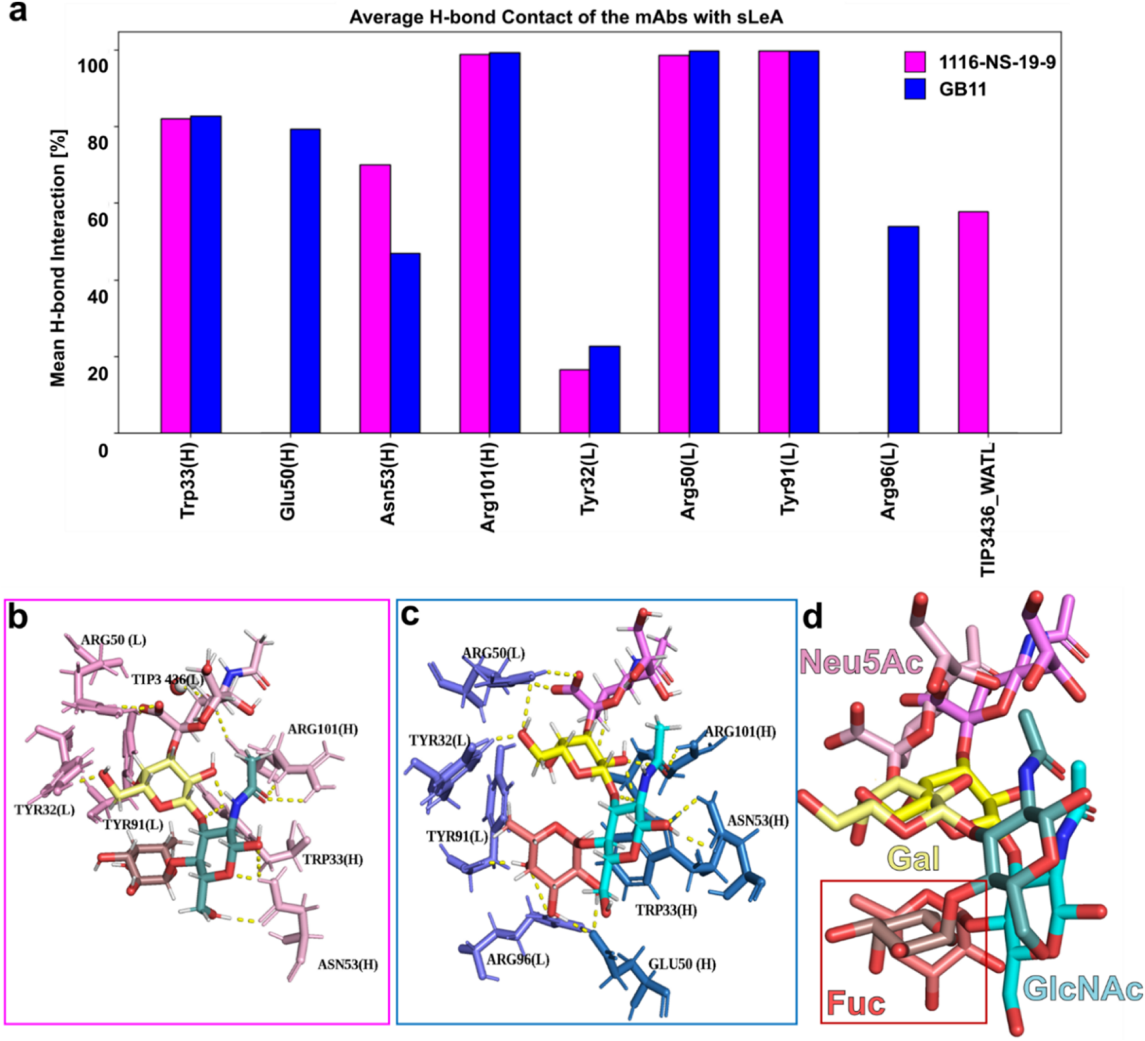
GB11 engages sLeA through distinct H-bonding and a different fucose orientation compared to 1116-NS-19-9. (a) Key binding-site residues of 1116-NS-19-9 (magenta) and GB11 (blue) involved in forming H-bond interactions with sLeA. The bars represent the stability of each hydrogen bond, shown as the average percentage of simulation time during which the bond was present. (b–c) Superimposed binding poses of sLeA at the lowest interaction energy in 1116-NS-19-9 (b) and GB11 (c). (d) In GB11, the Fuc unit adopts a rotated, vertical orientation that facilitates H-bond formation with Glu50­(H), Tyr91­(L), and Arg96­(L). This conformational shift emerges as a key factor contributing to the enhanced binding observed for GB11. In contrast, the horizontal or flat orientation observed in the Fuc, while binding to 1116-NS-19-9, does not promote stable H-bond formations with binding-site residues.

Consistent with these interactions, the strongest contacts between sLeA and the protein matrix involve the polar residues Arg101­(H) and Arg50­(L), followed by hydrophobic contacts with Trp33­(H), whereas Asn/Ile56­(H) contributes only weakly to binding. Minor ligand-induced SASA changes are observed for hydrophobic residues such as Ile56 and Val100, while a more pronounced SASA reduction is detected for Arg101, in line with its stronger interaction energy with sLeA (Figure S17, Table S4).

Finally, representative snapshots from the lowest-energy frames (Figure 4b,c) show that sLeA adopts a slightly shifted binding mode in both mAbs, with the Fuc unit rotated ∼90° in GB11, enabling stronger H-bonds with Tyr91­(L). In 1116-NS-19-9, Tyr91­(L) instead interacts with Neu5Ac. Overall, MD simulations indicate stable mAb–glycan complexes, with subtle binding-site differences causing minor ligand reorientation and H-bond variations, ultimately yielding higher sLeA affinity for GB11, consistent with our experimental results.

### STD NMR Spectroscopy Confirms Unique sLeA Binding Epitopes

To gain deeper insight into the differential recognition of sLeA by the three mAbs, we employed STD NMR spectroscopy. Binding was confirmed in all cases and further supported by negative control experiments conducted in the absence of mAbs (Figure S18).

A significant portion of the tetrasaccharide’s resonances fell within the same ppm range (3.2–4.2 ppm) and were, therefore, prone to strong overlapping, obstructing an unambiguous quantification of STD effects for specific resonances within this range. Hence, based on reported resonance values for sLeA derivatives,^43^ we carefully reassigned the resonances of sLeA under our experimental conditions using conventional two-dimensional NMR techniques (Table S4, Figures S19, S20). Accurate resonance assignment was essential to maximize both the interpretability and reliability of the subsequent STD NMR experiments. Of note, the acquired sLeA sample contained a mixture of α- and β-GlcNAc anomers, attributed to the presence of a free OH-group at the C1 position of GlcNAc. Accordingly, we refer to these forms as α- and β-anomers, respectively.

While in all three cases, dissociation of the complexes is sufficiently fast to bring about decent sensitivity in STD NMR measurements, 1116-NS-19-9 displays significantly stronger STD effects in terms of the absolute values compared to the other two mAbs (Figure 5a, Figures S21, S22), which is most likely caused by faster off-rates in line with its slightly lower affinity. Therefore, to avoid bias toward a certain NMR signal of sLeA, we normalized the STD effects relative to the sum of all of the STD effects separately for every mAb (Figure 5b). An alternative representation of the STD data, illustrating the absolute STD intensities referenced to the most intense ligand signal (set to 100%), is provided in Figure S23. The normalized STD effects mapped on the molecular structure of sLeA depict a rather similar distribution of the STD effects in the entire molecule for GB11 and HA8, with the strongest STD effects concentrated on the Gal, GlcNAc, and Fuc subunits (Figure 5c). In contrast, 1116- NS-19-9 exhibits a different pattern, with the strongest STD effects observed on the Neu5Ac, Gal, and GlcNAc subunits. Interestingly, the acetyl groups of Neu5Ac and GlcNAc feature higher STD effects for 1116-NS-19-9 compared to those of GB11 and HA8 (Figure 5c).

**Figure 5:**
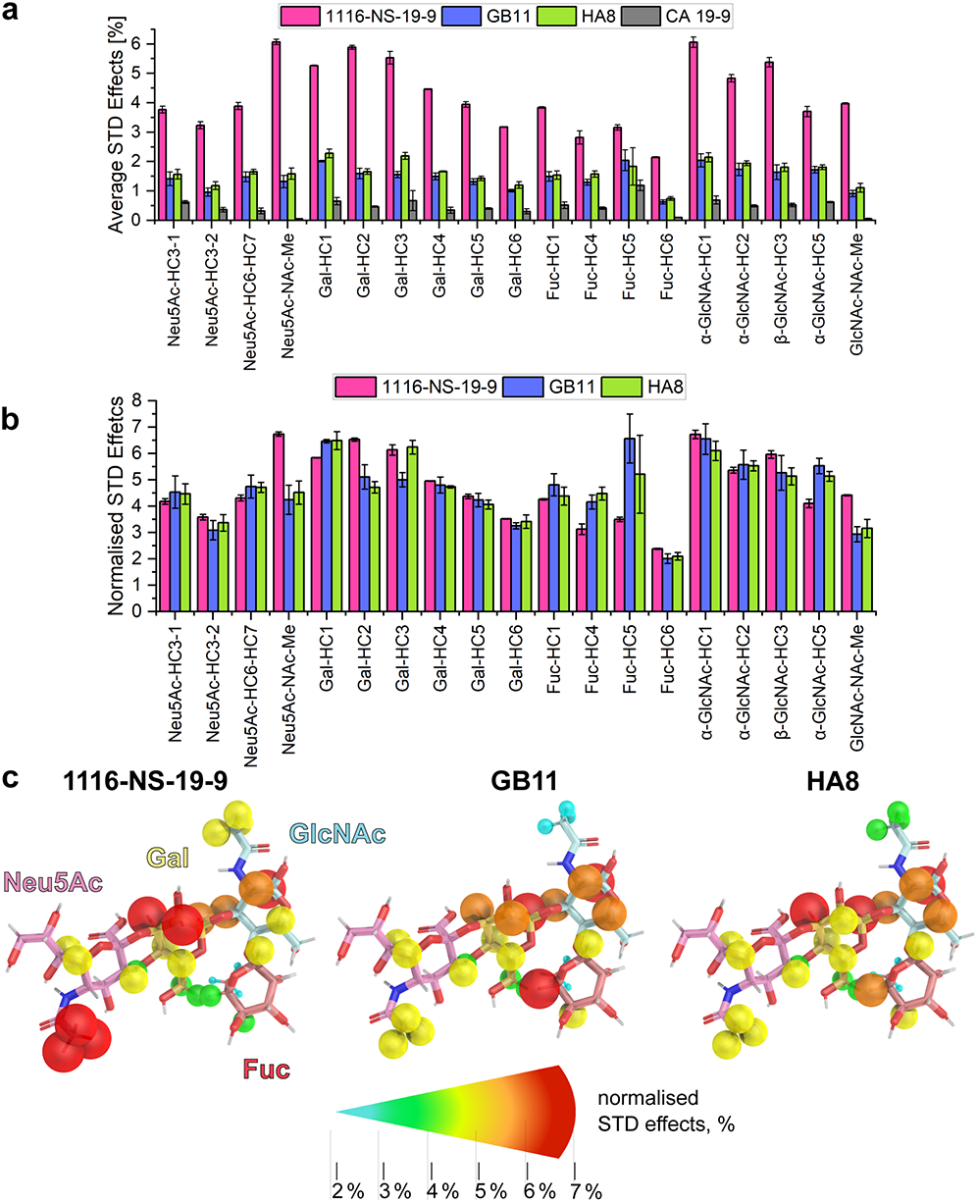
STD NMR reveals distinct sLeA binding epitopes for the three mAbs. (a) Average STD effects for sLeA hydrogen atoms from three independent STD NMR measurements with mAbs 1116-NS-19-9 (magenta), GB11 (blue), and HA8 (green). STD effects are expressed as a percentage of the respective sLeA signal without saturation. sLeA (gray bars) serves as a negative control STD NMR experiment in the absence of the mAbs. (b) Normalized STD effects relative to the sum of all STD effects for sLeA hydrogen atoms. (c) Normalized STD effects are mapped on the molecular structure of sLeA for each mAb, with increasing STD effects presented by color and size.

To further characterize the binding interfaces of HA8 and GB11, DEEP-STD NMR experiments were performed to probe the contribution to binding from aromatic vs. aliphatic amino acid residues.^44^ Indeed, in the case of both antibodies, slightly varying contributions are observed (see Figure S24). In the case of HA8, saturation of Neu5Ac is slightly stronger when saturating at the aliphatic chemical shift range, whereas saturation of GlcNAc is dominated by the contribution of aromatic amino acids. For Gal and Fuc, both contributions appear to be equally strong. In the case of GB11, there is, overall, less variation. Similar to HA8, Neu5Ac shows a stronger contribution from aliphatic residues. In contrast to HA8, contributions from aromatic residues for saturating GlcNAc are much less pronounced, and the sugar moiety with the strongest aromatic contribution is now Fucose. Apparently, in both HA8 and GB11, the Fucose methyl group receives the strongest saturation when saturating at the aliphatic chemical shift region.

## Discussion

Cancer patients with advanced and metastatic cancers often suffer from a poor prognosis and limited therapeutic options. However, effective mAbs targeting distinct cancer antigens, used for early cancer detection and as mono,^45–47^ adjuvant,^48,49^ or drug-conjugate^50,51^ therapy, drastically elongate patients’ survival. Our study focused on developing improved mAbs against sLeA, a tumor-associated carbohydrate antigen overexpressed in various cancers with poor survival rates.

To evaluate the effect of the antigen, we decided to use a classic immunization strategy with a glycoconjugate carrying synthetic sLeA. Two mouse-derived mAbs, GB11 and HA8, were isolated and characterized. These were compared to 1116-NS-19-9, an anti-sLeA mAb produced through immunization against the native, heterogeneous antigen, as presented on the human CRC cell line SW1116.^32,33^ A combined biophysical and bioinformatics’ study in comparison with *in situ* data gave a comprehensive overview of the antigen-binding process and provided clues for the improved specificity and affinity of GB11, compared to 1116-NS-19-9, especially to native sLeA, despite very high sequence similarity between both mAbs.

The *K*_D_ values obtained from both ITC and SPR measurements for GB11 and HA8 are consistent with each other (Table 1, Figure 1c), with a binding affinity within the low-micromolar range with synthetic sLeA in solution. SPR also shows a similar *K*_D_ for 1116-NS-19-9 (Figure 1c). Furthermore, all three mAbs exhibit no cross-reactivity toward glycans structurally similar to sLeA in glycan array assays (Figure S8b–d). Comparison of the variable regions of heavy and light chain sequences of GB11 and HA8 with those of 1116-NS-19-9 revealed remarkably high similarities in the variable regions (Figure 1a–b). The variations in the binding affinities of the mAbs, however, may be attributed to the small sequence differences in the CDR2.

Typically, germline Abs are recognized for their inherent polyspecificity due to heightened flexibility in their variable regions, owing to their limited mutational load in the germline V, D, and J genes.^52,53^ During the process of affinity maturation, Abs evolve through somatic hypermutations toward greater specificity and affinity for their target antigens,^54^ often by stabilizing the conformation and limiting the flexibility of the antibody’s paratope.^55,56^

Carbohydrate antigens, however, present a unique case. Because many tumor-associated glycans share high structural similarity with self-glycans, germline Abs recognizing these antigens are often intrinsically selective to avoid autoreactivity.^52,57–59^ This pre-existing selectivity may explain why affinity-matured Abs against such targets show a relatively minor sequence divergence from their germline precursors. In line with this, the high sequence similarity observed between GB11, HA8, and the earlier mAb 1116-NS-19-9 could reflect a shared origin from germline genes already predisposed to selectively recognize sLeA. This notion is supported by previous studies showing that germline antibodies targeting tumor-associated glycans can exhibit high selectivity with minimal affinity maturation.^57,58^ While our data do not directly confirm this mechanism, the observed similarities are consistent with such a model.

Despite their high sequence similarity, GB11 and HA8 exhibited more than a 25-fold higher MFI than 1116-NS-19-9 in synthetic glycan array assays, suggesting significantly stronger binding to immobilized, synthetic sLeA (Figure 1d). This difference may result from the fact that GB11 and HA8 were generated using a synthetic sLeA glycoconjugate for immunization, in contrast to 1116-NS-19-9, which was raised against native sLeA displayed on CRC cells. Therefore, we next aimed to investigate whether the observed binding performance would also be mirrored in the binding to sLeA in its native cellular context. Indeed, both GB11 and HA8 identified sLeA also on human PDAC and GAC tissues (Figure 2a–b). Moreover, GB11 demonstrated at least a 2-fold increase in binding to B16-FUT3+ cells compared to 1116-NS-19-9 (Figure 2d, Figure S9b).

To understand the origin of the observed binding differences as well as to gain insight into the role of sequence variation in the variable regions, we next analyzed the crystal structures, MD simulations, and STD NMR data of the mAb–sLeA complexes. The biological role of glycans is closely linked to their structural flexibility, particularly the geometries of glycosidic linkages and the puckering of individual monosaccharide rings.^60,61^ These intrinsic dynamics can influence the epitope presentation and antibody recognition. Despite their general flexibility,^62,63^ Lewis antigens like sLeA can adopt relatively rigid “closed” conformations, stabilized by an intramolecular CH···OH hydrogen bond between the Fuc and Gal residues.^64–67^ Although conformational transitions within sLeA are not directly captured in our data, transitions to alternative or “open” conformations have been observed in lectin complexes^68^ and proposed in computational studies.^60,61,69^

In light of these considerations, we next examined how sequence variation and glycan orientation influence the architecture of the binding site. We therefore began by analyzing the available crystal structures. Notably, specific binding pocket residues, such as Trp36 and Arg101, exhibited conformational changes upon ligand binding in the case of 1116-NS-19-9, as previously reported.^25^ Conversely, analysis of the apo- and holo-crystal structures of GB11 revealed that these same amino acids were already configured in the rotamer conformation that is observed in the case of 1116-NS-19-9 only upon binding to sLeA and not in the free state (Figure 3c–d). It should be noted that this finding is primarily based on comparing the apo structures that both have high resolution (1.51 Å in the case of 1116-NS-19-9, and 1.86 Å for GB11), and on the holo structure of 1116-NS-19-9 (1.55 Å). The holo structure of GB11 is only resolved to 2,80 Å, impeding the precise definition of the rotamer state of the side chain solely based on the crystal structure. However, the very high similarity of the binding mode is further corroborated by our MD simulations and, in particular, the careful preparation of the starting structure via redocking. The preorganization of GB11 mirrors the binding behavior observed in the mAb 5B1,^25^ which was also generated through immunization with synthetic sLeA conjugated to KLH.^26^

Additionally, we found differences in the binding pocket formed by the heavy chain, where Thr100 of 1116-NS-19-9 was substituted with the more hydrophobic Val100 in GB11 and HA8, potentially contributing to the observed variation in the rotamer conformation of Arg101 by creating a more hydrophobic binding pocket (Figure 3c–d). Indeed, some of the mutations were previously introduced in a directed evolution campaign to improve sLeA binding in 1116-NS-19-9,^25^ achieving affinity gains comparable to those measured for GB11 and HA8. Beyond the binding site, specificity can also be modulated through alterations distal to the paratope.^70–72^ Indeed, the comparison among GB11, HA8, and 1116-NS-19-9 revealed that mutations predominantly occur outside the core binding site (Figure 1a–b). In the case of GB11, some occur even within the structurally conserved region (Figure S3). Such distal mutations may further contribute to the enhanced affinity of GB11 and HA8 by indirectly stabilizing the paratope or by modulating the VH-VL domain spacing and orientation, a phenomenon already observed for other antiglycan Abs.^57,58,71,73^

To support our structural studies, comprehensive MD trajectory analyses of the sLeA–mAb complexes in the solution phase were performed. In both complexes, the glycans resided stably in the binding pockets after the equilibration phase. Notably, the sLeA–GB11 complex exhibited a slightly lower interaction enthalpy than sLeA bound by 1116-NS-19-9 (1.7 kcal/mol averaged over the last 10 ns). This tendency is corroborated by the predicted interaction energies that are also more favorable in the case of the GB11-sLeA complex (3.1 kcal/mol), underpinning the higher affinity of GB11.

The sLeA tetrasaccharide adopts a very similar conformation when binding to the mAbs 1116-NS-19-9 and GB11, and such a conformation is also seen in complex with 5B1.^25^ However, when comparing in detail the conformational space of sLeA docked in 1116-NS-19-9 and in GB11, we observed notable differences in the orientation of the Fuc unit (Figure 4b–d). When superimposing the sLeA tetrasaccharide of both complexes, Fuc in the GB11 complex adopts a vertical orientation compared to its orientation in the 1116-NS-19-9 complex. This likely plays an important role in promoting stronger H-bond interactions with the key residues of the binding pocket (Glu50­(H), Tyr91­(L), and Arg96­(L)). Conversely, in 1116-NS-19-9, Tyr91­(L) forms a H-bond interaction with Neu5Ac instead. According to our MD simulations, this altered orientation of the Fuc results in stronger H-bond formation and promotes binding contributions of all four glycan units, thus further corroborating the overall observed stronger binding affinity of GB11 toward sLeA.

It should be pointed out that these conformational changes and H-bonds are of a transient nature and reflect the dynamic situation at room temperature in solution. Noteworthy, also in the X-ray structure, corresponding to an averaged lowest-energy conformation in a frozen crystal, the electron density of the Fuc residue binding to GB11 is the lowest of all four monosaccharide building blocks. The conformation and position within the binding site are therefore less well-defined than those for the rest of the sLeA ligand and may indicate increased flexibility. That the shifted and rotated orientation of the Fuc moiety may be relevant for GB11′s higher affinity toward sLeA is corroborated by the results of our STD NMR experiments.^74,75^ The altered positioning of the Fuc unit as observed in the MD simulation of the GB11 complex would orient its hydrophobic face, comprising H3, H4, H5, and the CH_3_-group, closer toward the side chain of Ile56­(H). This could explain the stronger saturation of Fuc-H4 and −H5 in the presence of GB11, which is indicative of a hydrophobic interaction that, in turn, is likely to contribute to the higher affinity of sLeA toward GB11 compared to 1116-NS-19-9 (Figure 5a–c).

Interestingly, the MD simulations of sLeA in complex with 1116-NS-19-9 reveal a unique H-bond between Tyr91­(L) and the sialic acid moiety Neu5Ac. This is evidence for a stronger contribution of this part of the sLeA tetrasaccharide that is nicely reflected by increased saturation of the Neu5Ac-acetyl-CH_3_ group in the STD NMR experiments.

When comparing the saturation received by the individual carbohydrate units in the mAb complexes, the STD NMR data for 1116-NS-19-9 show the strongest saturation for Gal and GlcNAc, followed by Neu5Ac, with the weakest signal at Fuc (Figure 5, Figure S25). In contrast, GB11 and HA8 show more uniform saturation across all four carbohydrate residues (Figure 5, Figure S25), again highlighting the stronger engagement of Fuc, as observed in the MD simulations.

In order to obtain more detailed insight into the differential contributions to ligand saturation from aromatic vs aliphatic residues, we performed a characterization via the DEEP-STD NMR approach.^44^ (see Figure S24)

Overall, these results nicely support the proposed binding modes. In the case of GB11, most glycan units show slightly stronger STD effects when saturating in the aromatic region, consistent with the presence of one Trp and multiple Tyr residues located in the direct vicinity. Only the sialic acid exhibits slightly more contribution from aliphatic contacts, probably mediated by the side chains of Arg101­(H), Ala103­(H), and Val100­(H).

The fucose methyl group appears to be a special case. It shows the strongest DEEP-STD contribution when saturated at the aliphatic chemical shift region. However, to a large extent, this is likely brought about by directly saturating the methyl group that is close to the indole ring of Trp33­(H). Some additional contributions may come from Ile56­(H).

Interestingly, in the case of HA8, the strongest aromatic contribution is observed for the GlcNAc moiety. Most likely, this is caused by the mutation at position 56­(H), where HA8 possesses an asparagine instead of an isoleucine residue. Thus, in HA8, GlcNAc is mainly saturated via Trp33­(H), whereas in GB11, this is balanced by significant saturation through Ile56­(H).

We stress that the antibody glycan systems studied here are certainly not ideal objects to be characterized by the DEEP-STD approach. Their STD effects are overall rather weak. Furthermore, the whole binding pocket is lined by aromatic residues, and there are additional aromatic residues “in the second row” located somewhat more distant from the glycan ligand itself but close to the relatively few aliphatic groups with close contact to the ligand. Taking the large size of the antibody into account, which promotes very efficient spin diffusion, we can assume significant relay processes blurring the results from the DEEP-STD experiments.

The combined insights from X-ray crystallography, STD NMR, and MD simulations not only reinforced our experimental results regarding the elevated binding affinity but also provided additional detailed insights into the molecular recognition of each complex, including the key interactions of sLeA with each mAb. Moreover, it corroborated the conclusion that GB11 engages sLeA more extensively than does 1116-NS-19-9. While GB11 interacts with all four monosaccharides units, 1116-NS-19-9 primarily contacts Neu5Ac, Gal, and GlcNAc (Figures 4b–c, 5b–c and, Figures S1 and S21). Increasing the number of glycan residues in contact with the antibody binding site has been shown to enhance both specificity and affinity by promoting a more extensive interaction network and stabilizing a favorable energetic conformation of the glycan.^53^ Hence, GB11 is one example of gaining a stronger affinity by enhancing the interaction interface to the carbohydrate antigen. A similar interaction pattern has been described for 5B1, which also engages all four residues,^25^ suggesting that full epitope recognition is a recurring feature among high-affinity anti-sLeA antibodies.

In summary, our comprehensive analysis and previous studies^25,26^ suggest that the strategic use of synthetic glycans in immunization can lead to the development of antiglycan Abs exhibiting higher binding specificity and affinity toward their target. Furthermore, using novel characterization of sLeA binding by STD NMR, we unveiled key determinants that shed light on the way these Abs may achieve superior performance through preorganized binding sites that precisely recognize distinct glycan configurations and engage the carbohydrate ligand through a broader contact surface.

## Conclusion

In this study, we generated and characterized two monoclonal antibodies, GB11 and HA8, raised against synthetic sLeA, and compared them with 1116-NS-19-9, which is one of the most used mAbs for clinical diagnosis of pancreatic cancer. In solution-based assays using synthetic antigen, the new mAbs displayed low-micromolar affinities (SPR *K*_D_ ≈ 2–4 μM; ITC *K*_D_ 2.4–3.6 μM), which are in the same range as determined for 1116-NS-19-9, and X-ray crystallography on GB11 revealed overall a similar binding mode. Remarkably, in glycan array assays with immobilized synthetic sLeA, GB11 and HA8 exhibited >25-fold higher MFI than 1116-NS-19-9, and in a cellular context, GB11 showed enhanced apparent binding compared to 1116-NS-19-9 (*K*_D_ ∼51 nM vs. ∼101 nM), underscoring the influence of antigen presentation on antibody performance. Detailed analysis by X-ray crystallography, MD simulation, and STD NMR spectroscopy reveals a different epitope engagement among the three mAbs and provides a potential rationale as to how slight sequence differences and a structural preorganization of the binding site lead to improved antigen recognition observed in particular in a cellular context. While this study is focused on molecular and biophysical characterization, it may provide a foundation for future functional investigation and in vivo evaluation on the way toward improved antiglycan antibodies.

## Supplementary Material



## Notes

Material and methods; Successful conjugation of sLeA to CRM_197_; RNA extraction and RT-PCR, revealing the light chain of GB11 and HA8 is a κ-chain; GB11 shows greater variation in conserved regions; GB11 and HA8 exhibit similar thermal stability compared to 1116-NS-19-9; GB11 and HA8 exhibit similar affinity towards synthetic sLeA; Raw SPR sensorgrams show concentration-dependent sLeA binding to immobilized mAbs; fitted SPR data reveals *K*_D_ ranges in micromolar range for each mAb; Glycans printed on CodeLink–glass slide; The mAbs show a high specificity towards sLeA only; GB11 binds significantly stronger to sLeA expressing mouse melanoma cells; X-ray data collection and model refinement statistics; Glycosidic angles of sLeA in the bound state; SLeA binding stabilises GB11 and 1116-NS-19-9 complexes; GB11 binding site for sLeA with lowest interaction energy score; Chemical Shift Assignment of sialyl Lewis A; ^1^H NMR spectrum and assignment of sLeA; 1H-13C HSQC and assignment of sLeA; In the absence of a protein, the STD spectrum of sLeA exhibits minimal saturation; The STD spectra of sLeA demonstrate interaction with all three mAbs; The STD spectra depict the resonances involved in the interaction between sLeA and the respective antibody; GB11 and HA8 show more uniform STD NMR saturation across sLeA glycan units than 1116-NS-19-9; CH–π geometry of TRP33­(H)–glycan contacts in representative low-energy conformations; Average total interaction energy (electrostatic + van der Waals) of binding-site residues from the heavy and light chains of 1116-NS-19-9 and GB11; Difference in the average solvent-accessible surface area (ΔSASA) of residues within the ligand-binding pocket between the holo and apo states; Solvent-accessible surface areas (SASA) of selected side chains in the 1116-NS-19-9 and GB11 mAbs; STD NMR effects on sLeA glycan in the presence of GB11, HA8 and 1116-NS-19-9; DEEP-STD NMR factors (ΔSTD) for sLeA in the presence of HA8 and GB11 (PDF)

## ◆

Scojen Institute for Synthetic Biology, Dina Recanati School of Medicine, Reichman University, Herzliya, Israel

## Abbreviations

Ab/Abs: antibody/antibodies
CA 19-9: carbohydrate antigen 19-9 (equals sLeA)
CDR: complementarity-determining region
CRC: colorectal carcinoma
DAGR: Database of Anti- Glycan Reagents
Fab: fragment antigen-binding region
FDA: Food and Drug Administration
Fuc: fucose
FUT3: fucosyltransferase III
Gal: galactose
GAC: gastric adenocarcinoma
GlcNAc: N-acetylglucosamine
H-bond: hydrogen bond
IgG: immunoglobulin G
IHC: immunohistochemistry
ITC: isothermal titration calorimetry
*K*_D_: dissociation constant
mAb/mAbs: monoclonal antibody/antibodies
MD: molecular dynamics
MFI: mean fluorescence intensity
MM-PBSA: molecular mechanics Poisson–Boltzmann surface area
Neu5Ac: N-acetylneuraminic acid
NMR: nuclear magnetic resonance
PDAC: pancreatic ductal adenocarcinoma
*R*_g_: radius of gyration
RMSD: root-mean- square deviation
RMSF: root-mean-square fluctuation
RT-PCR: reverse transcriptase-polymerase chain reaction
sLeA: sialyl Lewis A (equals CA 19-9)
SPR: surface plasmon resonance
STD: saturation transfer difference
TACA: tumor-associated carbohydrate antigen
