# Supplementary material for: Integrative Approach to Develop and Characterize Antibodies against the Cancer-Associated Antigen Sialyl Lewis A (CA 19-9)
Source: JACS Au. 2026 Apr 15;6(4):2274–87. doi: 10.1021/jacsau.5c01596 (PMC13126176; doi:10.1021/jacsau.5c01596)
Supplement: Supplementary file 1 [file au5c01596_si_001.pdf]

Supplementary Information for the Publication:

An Integrative Approach to Develop and Characterise Antibodies Against the Cancer-Associated Antigen Sialyl Lewis A (CA 19-9)

Anika Freitag[1,2,‡], Sana K. Khilji[1,3,‡], Ruslan Nedielkov[2,‡], Shalini M. Kumar[4], Michael Krummhaar[1,3], Janine Arndt[5], Gustavo M. S. G. Moreira[6], Jost Lühle[1,3], Felix Goerdeler[1,3], Carsten Kamphues[5,7], Maria A. Mroginski[4], Christian Roth[1,3], Peter H. Seeberger[1,3], Heiko M. Möller[2,*], Oren Moscovitz[1,*]

[1]Department of Biomolecular Systems, Max Planck Institute of Colloids and Interfaces, 14476 Potsdam, Germany

[2]Institute of Chemistry, University of Potsdam, 14476 Potsdam, Germany

[3]Institute of Chemistry and Biochemistry, Freie Universität Berlin, 14195 Berlin, Germany

[4]Institute of Chemistry, Technische Universität Berlin, 10623 Berlin, Germany

[5]Chirurgisches Forschungslabor, Klinik für Allgemein- und Viszeralchirurgie, Charité -Universitätsmedizin, Campus Benjamin Franklin, 12203 Berlin, Germany

[6]Tacalyx GmbH, Magnusstr. 11, 12489 Berlin, Germany

[7]Abteilung für Chirurgie in Weißensee, chirurgische Onkologie / Tumorchirurgie, Park-Klinik Weißensee, 13086 Berlin, Germany

*Corresponding authors: heiko.moeller@uni-potsdam.de, oren.moscovitz@mpikg.mpg.de

EXPERIMENTAL SECTION

Conjugation of sialyl Lewis A and CRM_{197}. 3'-sialyl Lewis A- (sLeA) was purchased from GlycoUniverse GmbH. CRM_{197} was conjugated to sLeA following standard protocols [1,2]. Briefly, 1 eq. of sLeA was mixed with 10 eq. of homobifunctional bis(p-nitrophenyl) adipate in 300 µL DMSO + 25 µL pyridine + 10 µL triethylamine (TEA) and stirred for 3 h at 300 rpm, room temperature (RT). After lyophilisation, the glycan half ester was washed 3x with chloroform and 3x with dichloromethane until uncoupled linker was no longer detected in the wash fractions. Next, 70 eq. of washed glycan half ester were mixed with 1 eq. of CRM_{197} in 140 µL conjugation buffer (0.1 M sodium phosphate pH 8) and stirred for 24 h at 70 rpm, RT. The degree of loading (DOL) was assessed by MALDI in linear positive ion mode using a 2,5-DHAP matrix and an Autoflex Speed (Brucker Daltonics). Comparing spectra of unconjugated CRM_{197} and sLeA-CRM_{197}, we determined a DOL of 7.4 glycans/protein (Figure S1).

Mouse Immunisation. Equal volumes of sLeA-CRM_{197} (in sterile PBS) and aluminum hydroxide Al(OH)3 (Alhydrogel) were mixed overnight at 4°C to allow adsorption of the glycoconjugate to the adjuvant matrix. Five 6- to 8-week-old female C57BL6JRj mice were immunised subcutaneously with 1 µg sLeA (equal to 8.65 µg sLeA-CRM_{197}) per injection. The mice received the glycoconjugates 4 times on day 0, 14, 28, and 42, followed by a boost injection on day 68. Three days after the boost injection, the spleen were harvested. Animal experiments were performed by Hybrotec GmbH (Germany, Potsdam) and approved by the Landesamt für Arbeitsschutz, Verbraucherschutz und Gesundheit (LAVG) Brandenburg (Gesch-Z. 2347-A-34-1-2020). Experiments were performed according to the German law, following the regulations of the Society for Laboratory Animal Science (ALAS) and of the Federation of Laboratory Animal Science Associations (FELASA).

Monoclonal Antibody Development. Monoclonal antibodies were obtained via hybridoma technology from mouse splenocytes as previously described in Broecker et al.[1]. The selection of hybridoma clones was done by glycan microarray-assisted analysis [3]. After three subsequent subcloning steps, two hybridoma clones, GB11 and HA8, producing Immunoglobulin G1 (IgG1) exclusively binding to sLeA were recovered.

Hybridoma Culture. Hybridoma cells were stored at -80°C in RPMI + 10% FCS and 10% DMSO at a concentration of $2x10^6$/mL. For a new culture, cells were thawed at 37°C for 30 s before they were washed in ISF-1 medium with 10% FCS to remove DMSO. Cells were centrifuged at 300 g/RT for 5 min. The supernatant was removed and taken up afresh in 10 mL of ISF-1 + 10% FCS. The cells were cultured at 37°C in the presence of 5% CO2 and the FCS was gradually decreased over days until it was completely removed. Cells were then passaged every 2-3 days until they were expanded to 300 cm^2 flasks. They were then allowed to grow for 7-10 days until they died. The cultures were centrifuged at 1,200 g/RT for 20 min. The supernatant containing mAb was sterile filtered with a 0.2 µm filter and stored with 0.02% NaN3 at 4°C to avoid contamination.

RNA Isolation and Sequencing of the mAb GB11 and HA8. RNA was isolated from 10^7 hybridoma cells of either clone GB11 or HA8, using RNeasy MiniKit (Qiagen, Hilden, Germany). The cDNA of the variable domains was amplified via template-switch reverse transcription as described elsewhere [4]. Briefly, cDNA was synthesised using chain-specific primers for mouse IgG heavy chain, kappa light chain, and lambda light chain in the presence of a template-switch oligo (AAGCAGTGGTATCAACGCAGAGTACATrGrGrG, where r stands for RNA base) to add a 3' adaptor sequence for subsequent sequencing. Reverse transcription was performed using the SuperScript IV Reverse Transcriptase system (Invitrogen), which is capable of template switching. Subsequently, cDNA from each chain was amplified via PCR using chain-specific nested reverse primers that were modified with 5'-phosphates, along with an adaptor sequence-specific universal forward primer. Generated fragments were purified on a 2% agarose gel and blunt end ligated into the pCRZeroT plasmid, previously digested with SmaI (NEB). The pCRZeroT plasmid was a gift from Ken Motohashi (Addgene plasmid #120276; RRID: Addgene_120276 [5]). Ligation products were transformed into the Escherichia coli (*E. coli*) DH5alpha strain. Plasmid DNA from successfully transformed colonies was isolated and sequenced using the M13 standard primers to obtain variable domain sequences.

Monoclonal Antibody Purification. Fast protein liquid chromatography (FPLC) was performed at 4°C on an ÄKTApurifier UPC10 System (GE Healthcare; Uppsala, Sweden) operated with the UNICORN 5.11 software. A 5 mL Pierce Protein A/G Chromatography cartridge (Thermo Scientific; Rockford, IL, USA) was equilibrated with binding buffer (25 mM Sodium phosphate, 150 mM NaCl, pH 7.4) by passing through 10x column volumes (CV) through the cartridge. Up to 2 L of hybridoma supernatant containing the mAb was passed through the column overnight, at flow rates ranging from 0.5 to 1 mL/min. The cartridge was washed with 15xCV binding buffer followed by 10xCV of a mixture of 80% binding buffer and 20% protein A/protein G elution buffer (100 mM Glycine-HCl, pH 2.7). Finally, the resin-bound mAb was eluted with 15-20xCV 100% protein A/protein G elution buffer at a flow rate of up to 5 mL/min and collected in 5 mL fractions, containing a previously measured amount (approx. 155 µL) of protein A/protein G neutralisation buffer (1 M Tris-HCl, pH 9), for a final pH of 7.4. The presence of protein within the fractions was followed by UV absorption at 280 nm. The fractions containing eluted mAb were concentrated using a centrifugal Amicon® Ultra-4 filter (MWCO 30 kDa) (Merck Millipore; Tullagreen, Ireland) and further purified by size exclusion chromatography on the ÄKTApurifier with a HiLoad 16&600 Superdex 200 pg (S200) (Cytiva) in 1x phosphate-buffered saline (PBS, pH 7.4). All fractions were analysed by SDS-PAGE. The eluted fractions containing the mAb were concentrated using a centrifugal Amicon® Ultra-4 filter (MWCO 30 kDa), and the protein concentration was determined with a NanoDrop ND-1000 Spectrophotometer (Thermo Scientific; Waltham, MA, USA). The mAb was stored in 1xPBS containing 0.02% NaN_3 at 4°C.

Thermal Shift Assay. The mAbs GB11, HA8, and 1116-NS-19-9 were diluted with PBS to 0.15 mg/mL. Intrinsic fluorescence was recorded at 330 nm and 350 nm while heating the sample from 35 to 95°C at a rate of 3°C/min. For the data collection as well as calculation of the ratio of fluorescence (350/330 nm) and the inflection temperature Ti, the NanoTemper Tycho NT.6 (NanoTemper Technology GmbH, Munich, Germany) was used according to the manufacturer's instructions.

Isothermal Titration Calorimetry. ITC analyses were conducted on an iTC200 instrument (MicroCal). GB11, HA8, and 3'-sialyl Lewis A (BIOSYNTH Carbosynth, OS00745) were diluted in PBS buffer, pH 7.4. 300 µL of 12.5-20 µM mAb was loaded into the sample cell, while sLeA was titrated with the syringe via 18 injections. The first injection was of 0.4 µL, while the rest were of 2 µL each. The final molar ratio of mAb to sLeA was in both cases between 1:20 and 1:24. All measurements were conducted at 25°C. Data was analysed and fitted with NanoAnalyze Data Analysis (Version 4.1.0.1). For both mAbs, at least three repetitions were carried out and the final average values are provided with standard error of mean. Due to the financial constraints associated with purchasing the 1116-NS-19-9 in amounts needed to carry out triplicates, this mAb was not included as a positive control in our analysis. Instead, the glycan was titrated to 1x PBS buffer as a negative control.

Surface Plasmon Resonance. Binding experiments were carried out on a Biacore T100 instrument (Cytiva Life Sciences, Danaher Corporation) using the Biacore T200 control software. On a CM5 chip, the Mouse Antibody Capture Kit (Cytiva Life Sciences, Danaher Corporation) was used according to manufacturer's protocol to first immobilise ~3,000-5,000 response units (RU) of α-mouse IgG capture antibody on two flow cells, one of which was used for mAb immobilisation and the other as a 'blank'-immobilised flow cell for reference to compensate for unspecific binding of glycan to sensor chip surface and α-mouse IgG capture antibody. Immobilisation of mAbs and glycan binding assays were performed at 25°C in PBS. Approximately 400 RU of mAbs were captured at either a concentration of 50 µg/mL for IgG GB11 and HA8, or a 1:100 dilution factor for 1116-NS-19-9 (Thermo Fisher #MA5-12421). The surface contact time was 180 s with a flow rate of 30 µL/min and a stabilisation period of 60 s to allow any uncaptured mAb to be washed away. Following this, sLeA was run over the immobilised mAbs in 2 cycles with increasing concentrations ranging from 0.375 µM to 100 µM so that 10 different concentrations could be measured. The highest concentration of the first cycle was taken as the lowest concentration of the second, and both cycles were plotted on the same graph using "Single cycle kinetics". The parameters used were a contact time of 60 s and a dissociation time of 180 s at a flow rate of 30 µL/min. Flow cells were regenerated with 10 mM glycine-HCl pH 1.7 with a contact time of 120 s at a flow rate of 20 µL/min, and a stabilisation time of 300 s was added to ensure that the baseline returned to its original value and all secondary mAb molecules were removed. For all mAbs, 3 repetitions were carried out. Analyses were carried out using the Biacore T200 evaluation software 3.2. As the on- and off-rates were outside of the measurable ranges of the instrument, the K_D values were determined using a "steady-state affinity" model. Each replicate dataset for every mAb was normalised such that the highest recorded RU was set to 100%. The normalised binding responses were then plotted against the logarithm of sLeA concentrations. Curve fitting was performed using the "log(agonist) vs. response – variable slope" model in GraphPad Prism (v10.4.2, Windows), applying minimum and maximum constraints of 0 and 100. The resulting data were fitted to the following equation:

$$Y = Min + \frac{(Max - Min)}{1 + 10^{(Log(EC50 + X))*HillSlope}}$$

X: log of concentration; Y: Response, increasing as X increases; Minimum (Min) and Maximum (Max): Plateaus in same units as Y (Min = 0; Max = 100); logEC50: same log units as X; Hill Slope: Slope factor or Hill slope, unitless.

Glycan Array Assay. The synthetic glycans (0.1 mM) were printed in lab on glass slides as described elsewhere [6,7]. The arrays contained immobilised sLeA and structurally related glycans of the Lewis family such as LeA and B, LeX and Y, sLeX, as well as additional TACAs like sialyl Tn (sTn) and Tn. As negative controls, the glycan array included immobilised protein (100 µg/mL) such as a mouse IgG and CRM_{197} (Table S1, Figure S7). The printed slide was blocked with 50 µL of 3% BSA/PBS per well at 37°C. After 1 h incubation, the wells were washed once with 50 µL PBS and incubated for 1 h at 37°C/slight shaking with 50 µL of 5 µg/mL of either HA8, GB11 or 1116-NS-19-9 (OriGene Technologies, Inc., #CF190083) in 1% BSA/PBS. After the incubation of the primary mAb, the wells were washed twice with 50 µL PBS for 15 min at 37°C/slight shaking each. The secondary antibody Alexa FluorTM 635, goat α-mouse-IgG (H+L) (Invitrogen by Thermo Fisher Scientific) was added with a dilution of 1:500 in 1% BSA in PBS and incubated for 1 h at 37°C. The wells were then washed twice with 50 µL PBS for 5 min each. As a final wash, the 64-well grid was removed, and the slide was dipped several times into ddH2O in a 50 mL falcon. Once dried by centrifugation at 300 g for 5 min, the slide was directly scanned using a Glycan Array Scanner Axon GenePix® 4300A (Molecular Devices, LLC, San Jose, CA, USA). The binding was analysed using GenePix Pro7 (Molecular Devices, LLC).

Immunofluorescence Staining. Surgically resected tissue was fixed in neutral buffered 4% formaldehyde for 16-24 h and processed for paraffin embedding. After deparaffinisation of 4 µm tissue sections, antigen unmasking was performed using 10 mM sodium citrate buffer (pH 6.0) with 0.05% Tween for 60 min at 90°C. Paraffin sections were permeabilised with 0.5% Triton X-100 for 10 min and blocked with 5% normal goat serum and 1% BSA for 60-120 min. Incubation with primary mAbs was performed overnight at 4°C in blocking solution using either the commercial mouse mAb, 1116-NS-19-9 (1:100, Thermo Fisher #MA5-12421), or the newly generated mouse mAb, GB11 or HA8 (5 µg/mL), against sLeA, simultaneously with a rabbit mAb against Vimentin (1:250, clone EPR3776, Abcam #ab9254). After rinsing with PBS, sections were incubated for 60-90 min at 37°C in 1% BSA with 4′,6-diamidino-2-phenylindole (DAPI, 1:1,000) and the secondary mAbs goat anti-mouse Alexa FluorTM 594 (1:250, Thermo Fisher #A-11005) as well as goat anti-rabbit Alexa FluorTM 488 (1:500, Abcam #ab150081). The paraffin sections were embedded in ProTaqs® MountFluor and analysed using the confocal Laser Scanning Microscope 510 META (Zeiss).

Cell Culture. B16 and B16FUT3+ cells were kindly gifted by Prof. Dr. J. V. Ravetch[8]. The cells were grown in Dulbecco's Modified Eagle Medium (DMEM; PANTM Biotech), supplemented with 10% foetal calf serum (FCS, PANTM Biotech), 2 mM/mL L-glutamine (PANTM Biotech), 10 U/mL penicillin/10 µg/mL streptomycin (PANTM Biotech) at 37°C in the presence of 5% CO2. For B16 FUT3+, 500 µg/mL of Geneticin (GibcoTM) as a selection antibiotic were additionally added to the growth medium. For binding assays, cells were collected out of culture flasks using Trypsin/EDTA.

Flow Cytometry. 1 x 10^6 cells per sample were used. After centrifugation for 5 min at 300 g, the cell pellet was incubated with 5 µg/mL of either HA8, GB11, or 1116-NS-19-9 (OriGene Technologies, Inc., #CF190083) in PBS for 1 h at 25°C. For the titration binding assays, a concentration range of 0.155 ng/µL – 40 ng/µL for all mAbs was used. The cell pellet was then washed twice with PBS, after a centrifugation of 5 min at 300 g. Following the washing steps, the cells were incubated for 1 h with Alexa FluorTM 635, goat α-mouse-IgG (H+L) (Invitrogen by Thermo Fisher Scientific) (1:500 dilution in PBS). The samples were then washed three times with PBS, resuspended in 200 µL PBS and analysed by flow cytometry. The data was collected on a FACSCantoII (BD Biosciences, San Jose, CA, USA) and analysed with FlowJo (v10.8.1, FlowJo LLC, Ashland, OR, USA). First, intact cells were selected (FSC-A over SSC-A) and then gating was carried out for single cells (FSC-W over FSC-H). The binding of mAb was examined in histograms of the APC channel. The apparent K_D values were determined by normalising the measured mean fluorescence intensity (MFI) to the highest MFI observed across five independent experiments for each antibody. The normalised MFI values were then plotted against the logarithmic concentrations of the mAbs. Curve fitting was performed using the "log(agonist) vs. response – variable slope" model in GraphPad Prism (v9.3.1, Windows), with the bottom and top constraints set to 0 and 100, respectively. The EC50 value, defined as the mAb concentration required to elicit half-maximal response, was used to determine apparent binding affinity. The fitted data were modelled using the following equation:

$$Y = Min + \frac{(Max - Min)}{1 + 10^{(Log(EC50+X))*HillSlope}}$$

X: log of concentration; Y: Response, increasing as X increases; Minimum (Min) and Maximum (Max): Plateaus in same units as Y (Min = 0; Max = 100); logEC50: same log units as X; Hill Slope: Slope factor or Hill slope, unitless.

Ficin Digestion. For GB11 and HA8, after purification, the eluted and concentrated mAb was injected onto an S200 column for buffer exchange to 100 mM sodium citrate/6.36 mM EDTA pH 6. Once concentrated, 4.39 mg/mL end concentration cysteine was added and incubated overnight with Ficin immobilised on agarose beads. The Fab product from the digestion was purified from the mixture with a 5 mL Pierce Protein A/G Chromatography cartridge (Thermo Scientific; Rockford, IL, USA) and buffer exchanged to the crystallisation buffer 10 mM Tris-HCl pH 7, 150 mM NaCl with the S200 column.

Crystallisation, Data Collection, and Structure Solution. Crystals of Fab fragments were grown using vapour diffusion in a hanging drop. Initially, 4x 96-well plates were set up with various crystallisation conditions. Sitting drops of 0.2 μL were added to the wells using an Oryx4 pipetting robot (Douglas Instruments). The plates were stored at RT until crystals were seen. The conditions giving the best crystals were set up in 48 well plates (condition 1: 2.06 M DL-malic acid pH 7.0, 2 mM citric acid pH 3.5, 0.5% PEG3350; condition 2: 1.6 M sodium malonate pH 7, 30 mM Hepes pH 7.0, 8.3% PEG3350). At a concentration of 12.4 mg/mL and 9.8 mg/mL, 2 μL of Fab fragments of GB11 or HA8, respectively, were mixed in a 1:1 ratio in the two conditions mentioned above. Crystals typically appeared within a week, and emerging crystals were fished. HA8 aggregated in the preliminary crystallisation steps and did not give crystals. Apo crystals of GB11 were directly frozen in liquid nitrogen, while holo crystals were incubated for seven days in mother liquor supplemented with 5 mM sLeA and then frozen in liquid nitrogen.

Diffraction data were collected at Berlin BESSY II, beamline 14.1 and 14.2 at 100 K. Data were processed with Xia2/DIALS [9–11], and the structure of GB11 without ligand was solved by molecular replacement with coordinates of 1116-NS-19-9 Fab fragment (PDB ID 6XTG) using MR Phaser [12]. The structure of the GB11-Sialyl Lewis A complex was solved using the refined structure of free GB11 as a search model. Structures were refined using Refmac5 [13] followed by iterative model-building cycles using Coot [14]. Restraints for sugars were created using Privateer [15]. Once refinement statistics converged, PDBredo with paired refinement [16] was used to determine whether data of higher resolution shells should be used. The resulting model was once more refined using Coot and Refmac5 as described previously. The relevant data collection and refinement statistics can be found in Table S2. Figures were created using CCP4mg.

Energy Minimisation and Molecular Docking. Computational studies were carried out to analyse the structural models of mAb-sLeA ligand complexes for GB11 and the well-characterised 1116-NS-19-9 as reference. Although crystal structures of both holo mAbs are available (PDB IDs: 6XTG for 1116-NS-19-9 and 9I6Q for GB11), the relatively low resolution of the GB11-sLeA complex (3.02–2.80 Å, PDB ID: 9I9H) necessitates additional refinement using molecular modelling techniques, including geometry optimisation and ligand docking. To evaluate the accuracy of the docking protocol, the sLeA ligand was redocked into the apo 1116-NS-19-9 antibody for comparison. The initial heavy-atom coordinates were obtained from the crystallographic structures mentioned above. Missing hydrogen atoms were added using PyMOL [17]. Owing to the poorly defined ligand electron density in the GB11 structure at 3.02 Å resolution, preliminary unrestrained molecular dynamics (MD) simulations resulted in structural instability. Consequently, the initial bound pose was rebuilt by minimising ligand–protein strain and repositioning the ligand within the known binding pocket using a constrained docking protocol, in which the search space was restricted to the experimentally identified pocket. Validation of the docking approach was achieved by redocking the ligand from the higher-resolution 1116-NS-19-9 complex, which successfully reproduced the crystallographic binding mode with an RMSD of 0.01 Å relative to the crystal structure (PDB ID: 6XTG).

As both mAbs share a high sequence similarity, we expected both models to entertain similar H-bond interaction networks. The 2D LigPlots in Figure S10, generated from the top-ranked tetrasaccharide poses of 1116-NS-19-9 and GB11, illustrate key interactions, with predicted binding affinities of -7.9 kcal/mol and -7.6 kcal/mol, respectively [18]. In both complexes, as detailed in the LigPlot analysis [19], sLeA forms H-bonds with key residues, including Arg101(H) (heavy chain), Arg53(L), and Arg96(L) (light chain). Additional interactions include H-bonds with Trp33(H), Asn53(H), Arg50(L), and Tyr91(L) for sLeA-1116-NS-19-9, and with Glu50(H) and Tyr32(L) for sLeA-GB11.

In case of the GB11-sLeA complex, the ligand coordinates were extracted separately from the mAb structure. Both ligands and mAb were solvated in explicit TIP3P water [20] within cubic boxes of 107 × 107 × 107 Å^3 (for GB11) and 39 × 39 × 39 Å^3 (for sLeA). The systems were then neutralised with Na^+ and $Cl^−$ counterions, and CHARMM-GUI tools were used for solvation and ion placement [21]. To optimise the structures of the solvated sLeA and apo GB11 models, energy minimisation was performed using the conjugate gradient algorithm [22,23] and the CHARMM36 force field [24]. Minimisation included 10,000 steps for GB11 and 5,000 steps for sLeA executed in NAMD (Nanoscale Molecular Dynamics, version 2.13) [25,26]. The sLeA tetrasaccharide ligand was modelled as a chain of four monosaccharides: N-Acetylglucosamine (GlcNAc), Galactose (Gal), Fucose (Fuc), and sialic acid (N-Acetylneuraminic acid (Neu5Ac)). For non-bonded interactions, a cutoff distance of 12.0 Å was applied, with a smoothing function spanning 10–16 Å. Pair-bonded atoms were excluded from non-bonded interactions, and the atom pair list for non-covalent interaction evaluations was updated every 20 steps.

Structural models of the holo GB11 and holo 1116-NS-19-9 mAbs were generated using rigid docking algorithms in AutoDock Vina [27,28], version 1.5.7. For holo GB11, docking was performed using the energy-minimised coordinates of isolated sLeA and apo GB11, while for holo 1116-NS-19-9, the ligand and mAb coordinates were directly obtained from the crystal structure. During this process, the mAbs were kept rigid, while the sLeA ligand remained flexible, allowing it to adapt to the conformation of the binding cavity. AutoDock tools (ADT) were used for preparing both mAbs and ligand files for docking. The grid box for 1116-NS-19-9 model was constructed around the ligand binding site residues, involving Asp31, Trp33, Asn53, Gly55, Asn56, Arg101 and Ala103 of the heavy chain (H) and Tyr32, Tyr49, Arg50, Tyr91 and Arg96 of the light chain (L). The dimension of the grid box in this model was set to 60 x 60 x 60 Å^3 (xyz) with a grid space of 0.375 Å. In the case of the GB11 model, the grid box was built around the key residues (Asp31, Trp33, Glu50, Asn53, Ala55, Ile56, Arg101 and Ala103) of the heavy chain and the Tyr32, Tyr49, Arg50, Tyr91 and Arg96 residues of the light chain. The grid box size of GB11 was set to 64 x 64 x 64 Å^3. Setting the grid boxes as described ensured that all sequence deviations among the binding site residues (K54Y, G55A and N56I) were included. Binding modes (poses) were ranked based on AutoDock Vina's binding affinity score. The highest-ranked pose was carefully inspected and selected as the starting structure for MD simulations.

A root mean square deviation (RMSD) of less than 0.01 Å between the heavy-atom coordinates of the crystal structure of the 1116-NS-19-9–sLeA complex and those obtained from the redocked model confirmed the reliability of the docking protocol and the predicted affinity scores.

Classical Molecular Dynamics Simulation. As a next step, extensive molecular dynamics (MD) simulations were performed [29]. To this end, the top ranked, stable docked sLeA complexes of 1116-NS-19-9 and GB11 were imported into NAMD version 2.13 [25,26]. Additionally, the apo forms of 1116-NS-19-9 and GB11 were simulated as reference systems to identify structural changes induced by ligand binding.

The MD protocol comprised of three major steps: energy minimisation, thermal equilibration, and production run. The CHARMM-GUI solution builder module was used for building the system with the rectangular box type and cubic crystal type of size 112 x 112 x 112 Å^3 for 1116-NS-19-9 and 109 x 109 x 109 Å^3 for GB11 [21]. The initial energy minimisation step consisted of 10,000 steps applying the same parameters as described above. During thermal equilibration, the systems were gradually heated from 0 K to 300 K using the NVT ensemble over 250 ps. A Langevin thermostat with a small friction constant of 1 ps^{-1} was applied to control and maintain a constant temperature [30]. During this phase positional restraints were applied to the protein backbone to prevent structural distortions while allowing solvent and counterions to equilibrate. Next, the equilibration

was continued with the production run under NPT ensemble for 200 ns maintaining a pressure of 1.013 bar using Nose-Hoover Langevin piston barostat with an oscillation period of 50 fs and an oscillation decay time of 25 fs [31,32]. To improve statistics of MD trajectory analyses, three replicates of 200 ns MD simulations were performed for the 1116-NS-19-9-sLeA and GB11-sLeA complexes, starting at randomly varied initial velocities.

Saturation Transfer Difference Nuclear Magnetic Resonance Spectroscopy. After purification, GB11 and HA8 were transferred to a D_2O-based PBS buffer (pH 7.4) through repeated ultrafiltration employing Amicon Ultra-4 devices (MWCO 30 kDa, Merck Millipore; Tullagreen, Ireland) until the H_2O-based buffer was sufficiently replaced (under 0.1% residual water). The lyophilised, commercial 1116-NS-19-9 mAb was resuspended in D_2O-based PBS buffer and trehalose, which was an additive present in the mAb sample, was removed via ultrafiltration. Therefore, the number of ultrafiltration steps for 1116-NS-19-9 was significantly higher than for GB11 and HA8. The 3'-sialyl Lewis A (BIOSYNTH Carbosynth, OS00745) was added from a 20 mM stock solution in the same D_2O-based PBS buffer (pH 7.4) to a final concentration of 200 μM. The mAb concentration was set to 6.7 μM. The resulting sample was transferred to a 3 mm NMR tube and measured immediately. Negative control samples without mAb were prepared identically, replacing the mAb solution with D_2O-based PBS (pH 7.4). The sample for the resonance assignment of sLeA was prepared by dissolving the compound in D_2O-based PBS buffer (pH 7.4) to a concentration of 13.7 mM.

NMR measurements were performed on a Bruker Avance III 600-MHz spectrometer equipped with an N2-cooled cryogenic 5-mm TCI-H/C/N-triple resonance probe at 300 K. Resonances of sLeA were assigned from a suite of 2D correlation NMR experiments, namely ^{1}H-^{13}C-HSQC, ^{1}H-^{13}C-HMBC, ^{1}H-^{1}H-TOCSY, ^{1}H-^{1}H-COSY and ^{1}H-^{1}H-NOESY. 1D saturation transfer difference (STD) NMR experiments were acquired using the standard pulse sequence "stddiffesgp.3" from the Bruker library [33]. Water suppression was achieved by excitation sculpting using an 8 ms square pulse [34]. On-resonance saturation of the proteins was achieved by a train of low power Gaussian-shaped pulses at 8.25 ppm with a total saturation time of 2 s. The off-resonance frequency was set to 30 ppm. On- and off-resonance spectra were acquired in an interleaved manner to minimise subtraction artefacts, of which 23,552 transients were collected. The on-resonance spectrum was subsequently subtracted from the off-resonance spectrum to yield the difference spectrum that was analysed and quantified. All spectra were acquired, processed and analysed with the software TopSpin (Bruker; v3.6: acquisition; v4.1: processing and analysis). The intensity of the STD effect was quantified by scaling down the off-resonance spectrum to match the intensity of the corresponding signal in the difference spectrum. All measurements were performed in triplicates to allow for statistical analysis. The amounts of the commercial 1116-NS-19-9 needed to carry out triplicates for this assay were beyond our financial means. Therefore, the same sample of 1116-NS-19-9 was measured three times.

The Differential Epitope Mapping (DEEP-STD) experiment was performed on the same HA8 and GB11 STD-NMR samples, for which two identical data sets were acquired that differed only in their irradiation frequency (6.69 ppm and -0.74 ppm, respectively). The results were analysed as described by Monaco et al. [35] The results are shown in Figure S25.

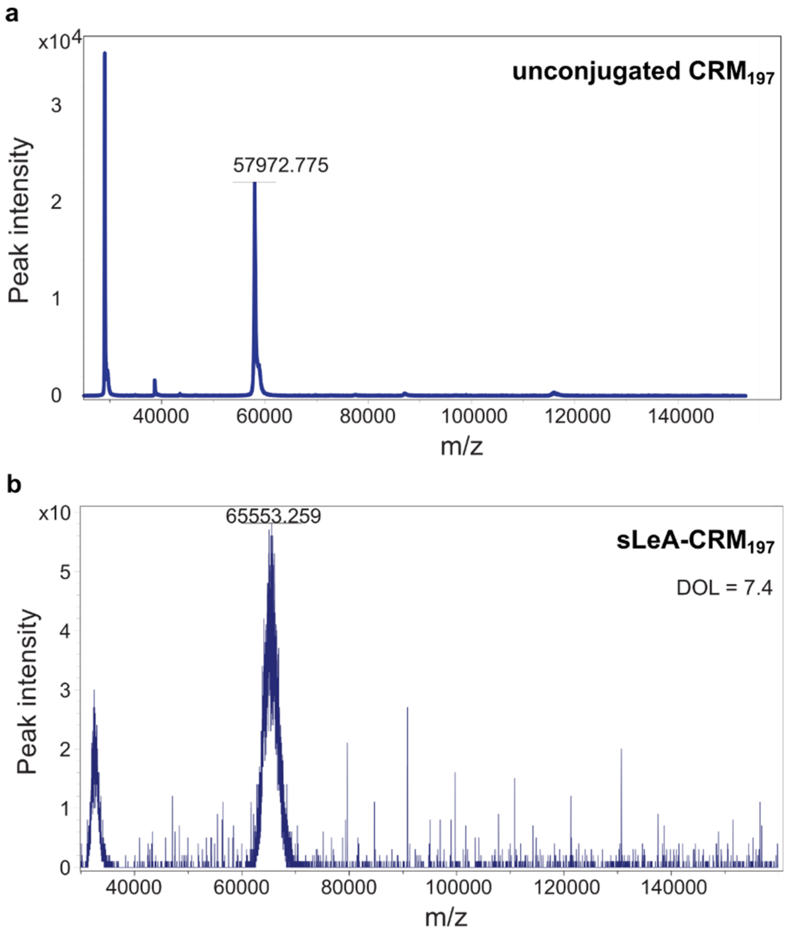

Figure S1| Successful conjugation of sLeA to CRM_{197}. *MALDI-TOF spectra of unconjugated CRM_{197} (**a**) and CA 19-9 conjugated to CRM_{197} (**b**), depicting a loading of approximately 7.4 sLeA molecules/protein.*

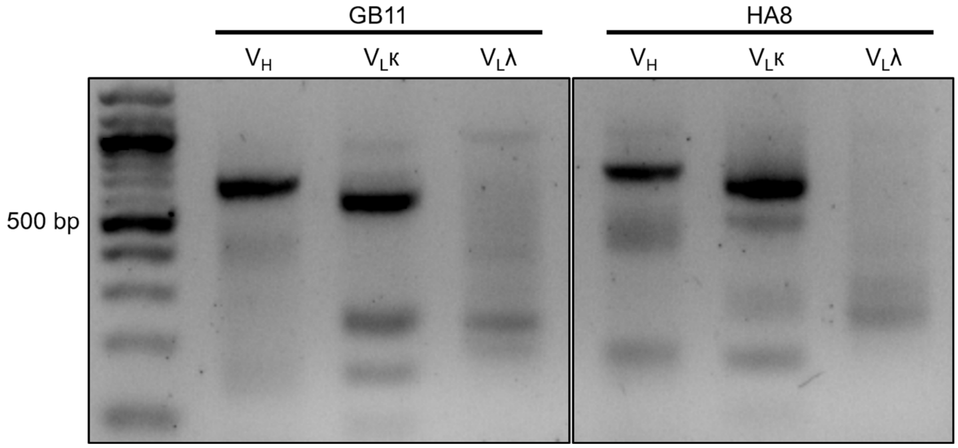

Figure S2| RNA extraction and RT-PCR, revealing the light chain of GB11 and HA8 is a κ-chain.

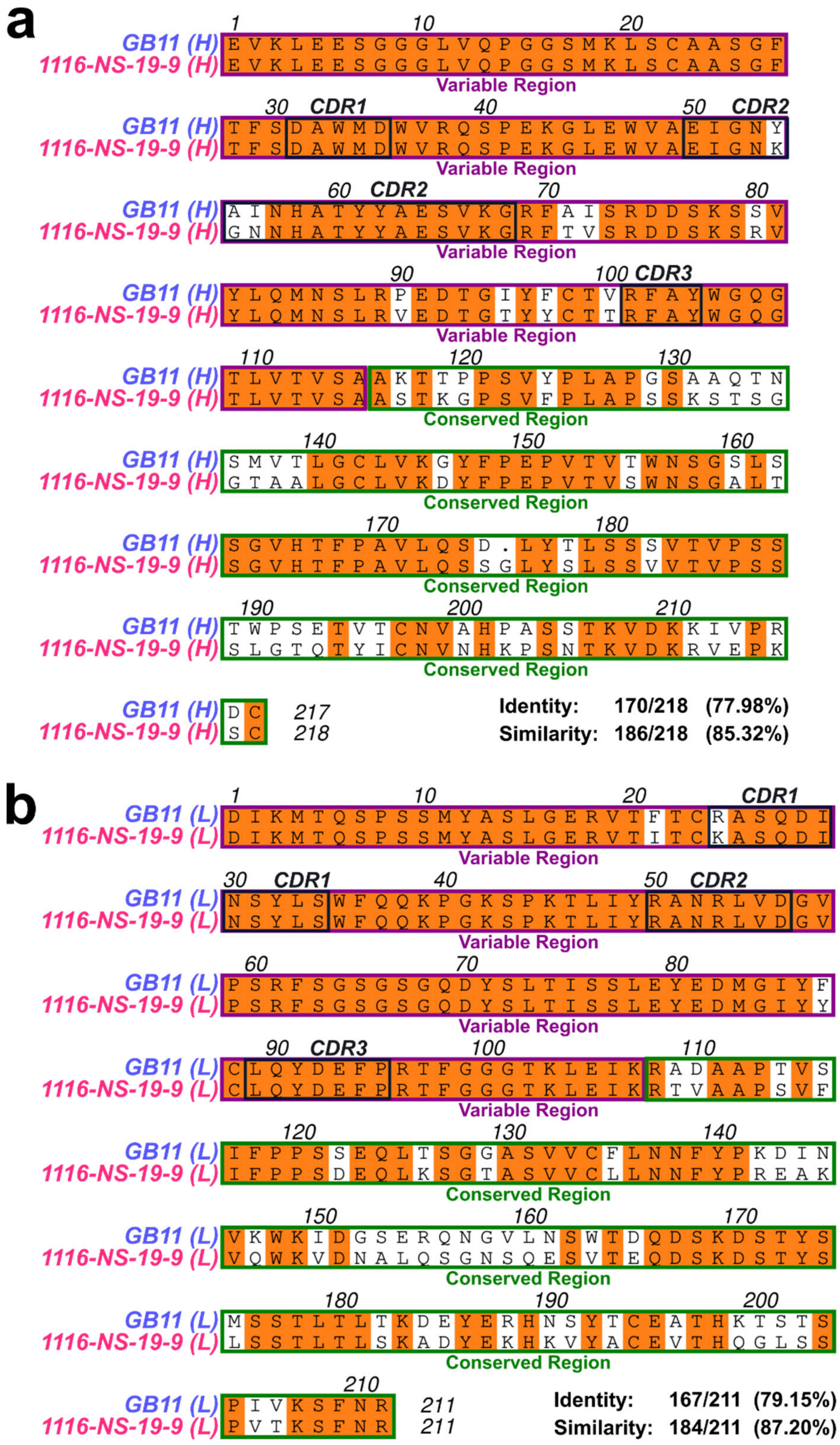

Figure S3| GB11 shows greater variation in conserved regions than in variable regions compared to 1116-NS-19-9. *The sequence alignments of heavy (**a**) and light chains (**b**) of the commercial mAb 1116-NS-19-9 (PDB ID: 6XTG) compared to the novel mAbs GB11 (PDB ID: 9I9H). The alignment is depicting the variable region (purple) as well as the conserved region (green) of the heavy and light chain respectively. The CDR regions are depicted in black. At the end the sequence identity and similarity are displayed.*

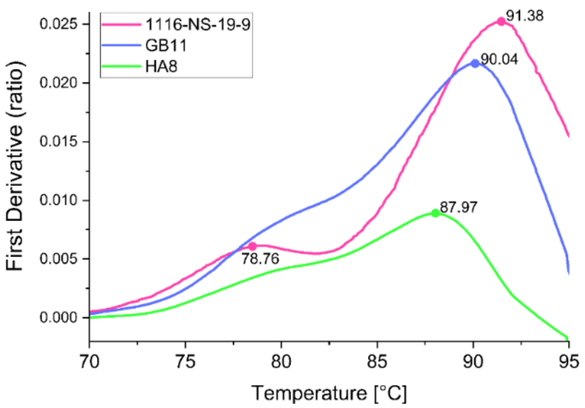

Figure S4| GB11 and HA8 exhibit similar thermal stability compared to 1116-NS-19-9. *Intrinsic fluorescence was recorded at 330 nm and 350 nm while heating the sample from 35 to 95°C at a rate of 3°C/min. Here, the first derivative of the 330/350 nm ratio is shown. The dots depict the melting points T. 1116-NS-19-9 reveals two melting points, called T1 and T2.*

a

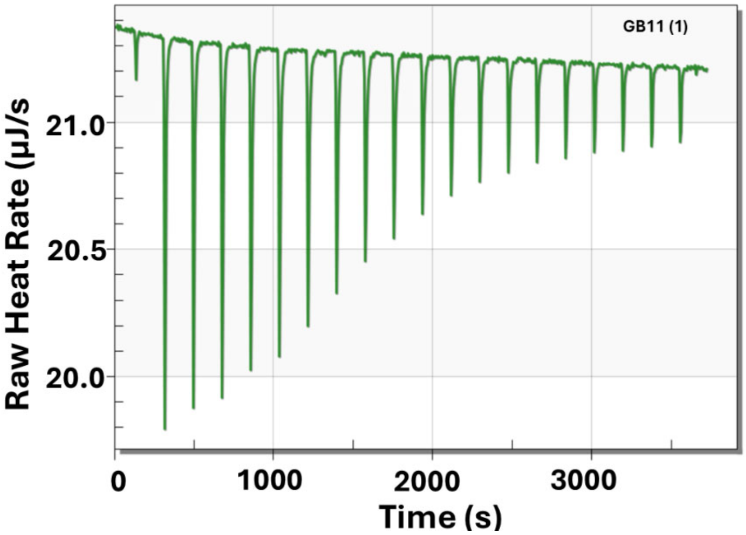

b

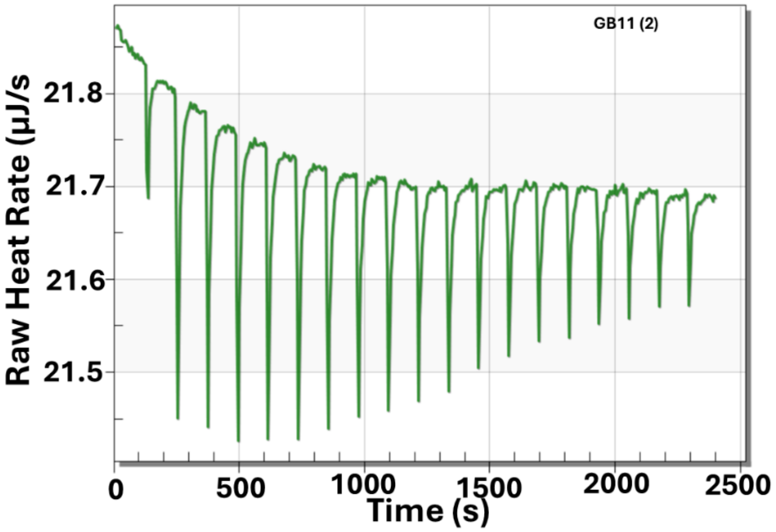
GB11 (2)
Raw Heat Rate (µJ/s
21.8
21.7
21.6
21.5
0
500
1000
1500
2000
2500
Time (s)

c

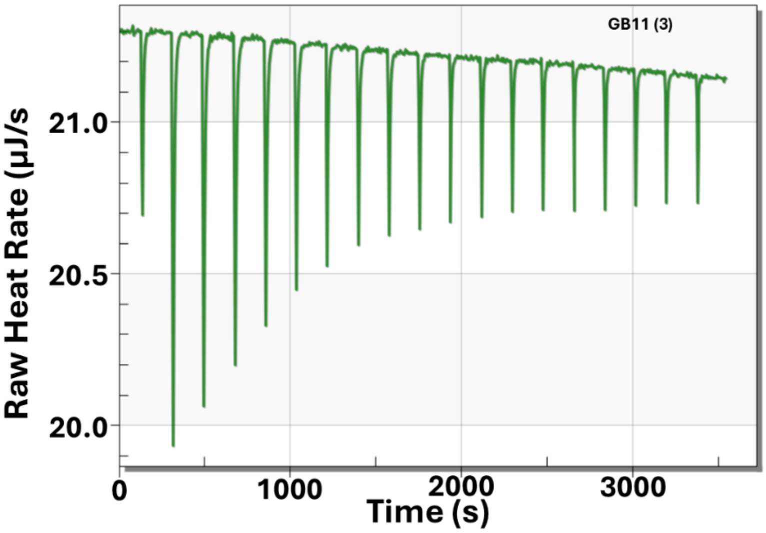
GB11 (3)
Raw Heat Rate (µJ/s
21.0
20.5
20.0
0
1000
2000
3000
Time (s)

d

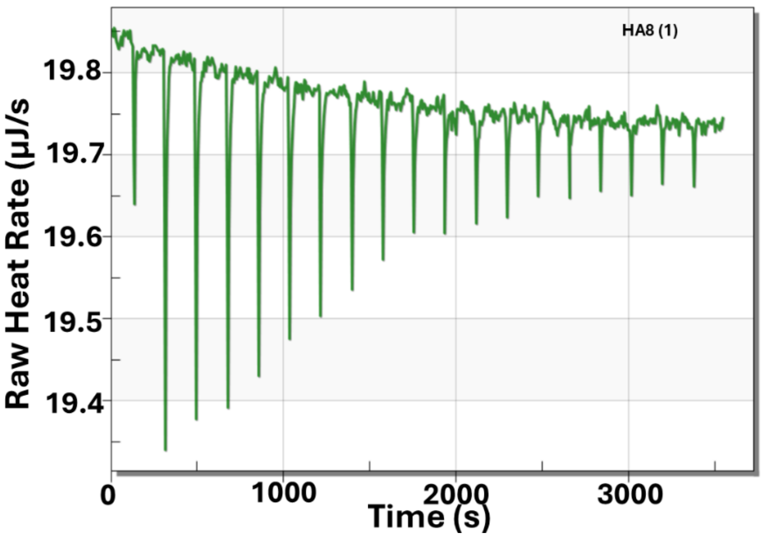
HA8 (1)
Raw Heat Rate (µJ/s)
19.8
19.7
19.6
19.5
19.4
0
1000
2000
3000
Time (s)

e

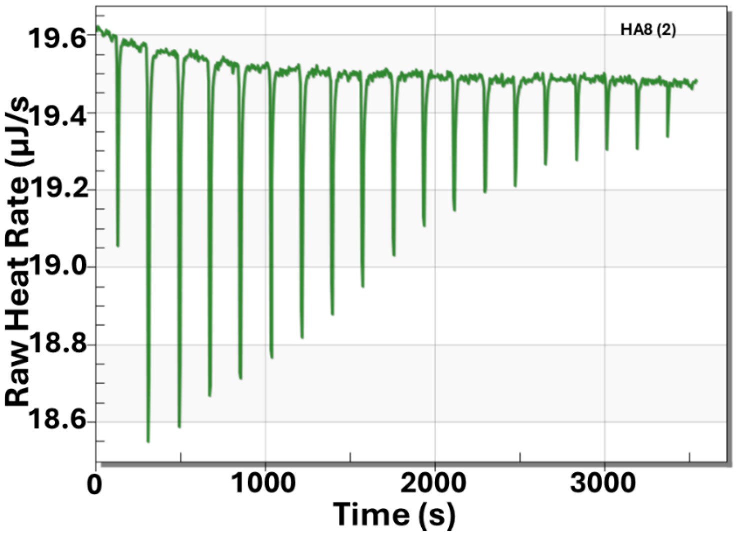
HA8 (2)
Raw Heat Rate (µJ/s)
19.6
19.4
19.2
19.0
18.8
18.6
0
1000
2000
3000
Time (s)

f

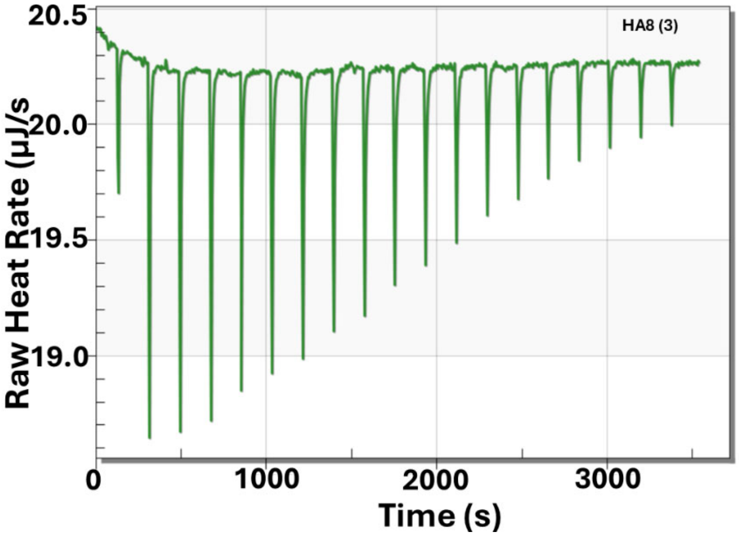

g

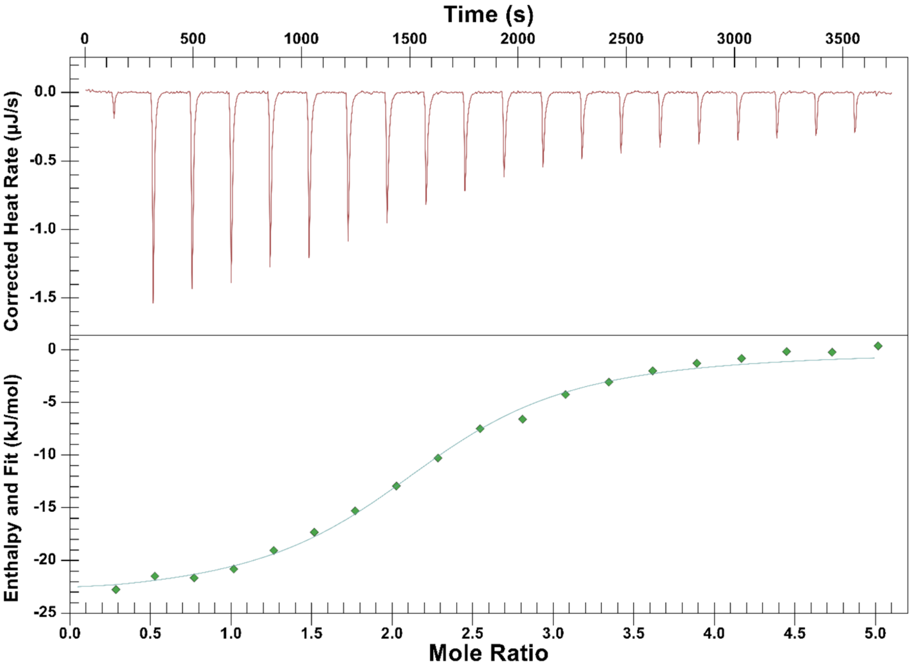

Figure S5| GB11 and HA8 exhibit similar affinity towards synthetic sLeA. *Isothermal titration calorimetry (ITC) of GB11 and HA8 with sLeA. The mAb was in the cell, while sLeA was injected. (**a-f**) Replicate raw thermograms in kJ per mol for each mAb are shown. (**g**)Fitted enthalpy (kJ/mol) with corrected heat rate (µJ/s) for GB11 (1) (figure a) as an example.*

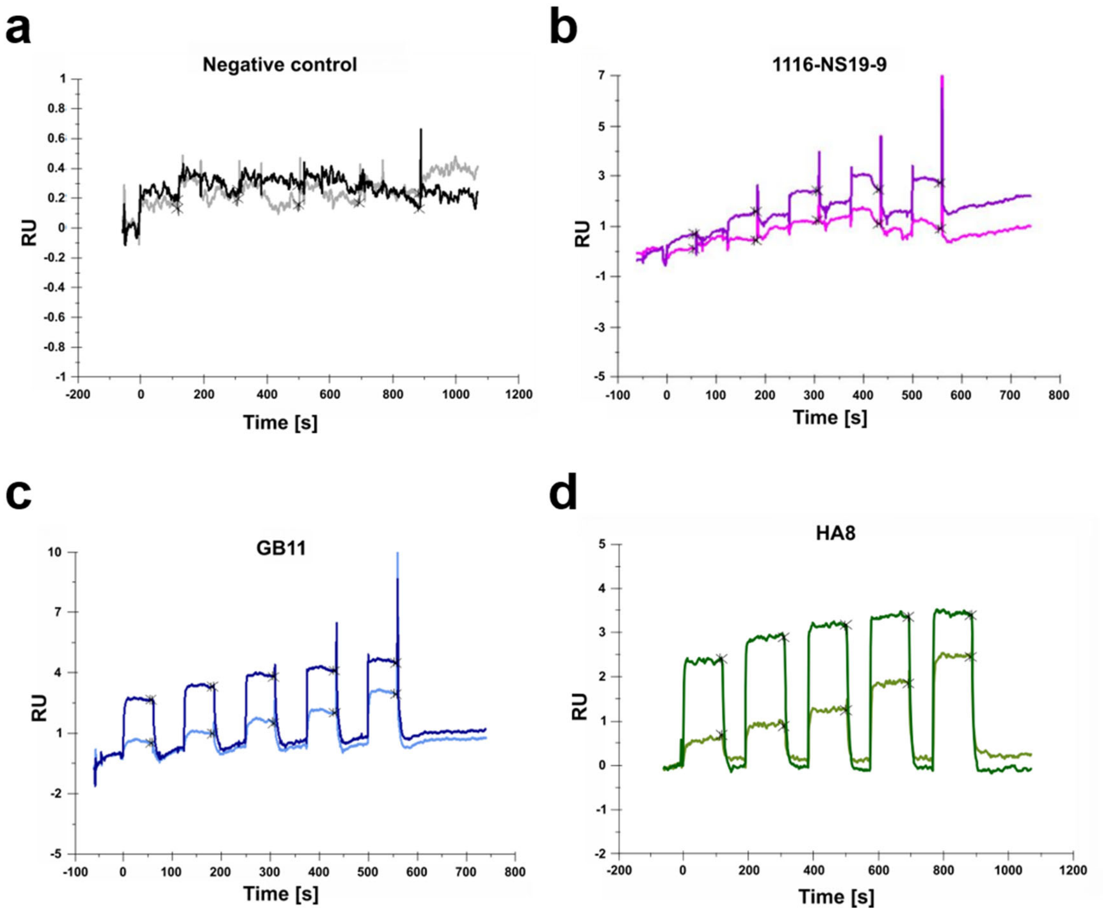

Figure S6| Raw SPR sensorgrams show concentration-dependent sLeA binding to immobilised mAbs *Surface plasmon resonance (SPR) measurements were performed using immobilised mouse monoclonal antibodies (mAbs) and synthetic sLeA as analyte. (**a-d**) Representative raw sensorgrams from one replicate per mAb are shown. Response units (RU) were recorded over time in seconds (s) across increasing concentrations of sLeA. Lower concentrations are shown in lighter shades of colour, higher concentrations in darker shades. The anti-mouse IgG from the immobilisation kit, used as a negative control is shown in grey (**a**). 1116-NS-19-9 is shown in magenta (**b**), GB11 in blue (**c**), and HA8 in green (**d**). RU values were recorded at the indicated time points after washing.*

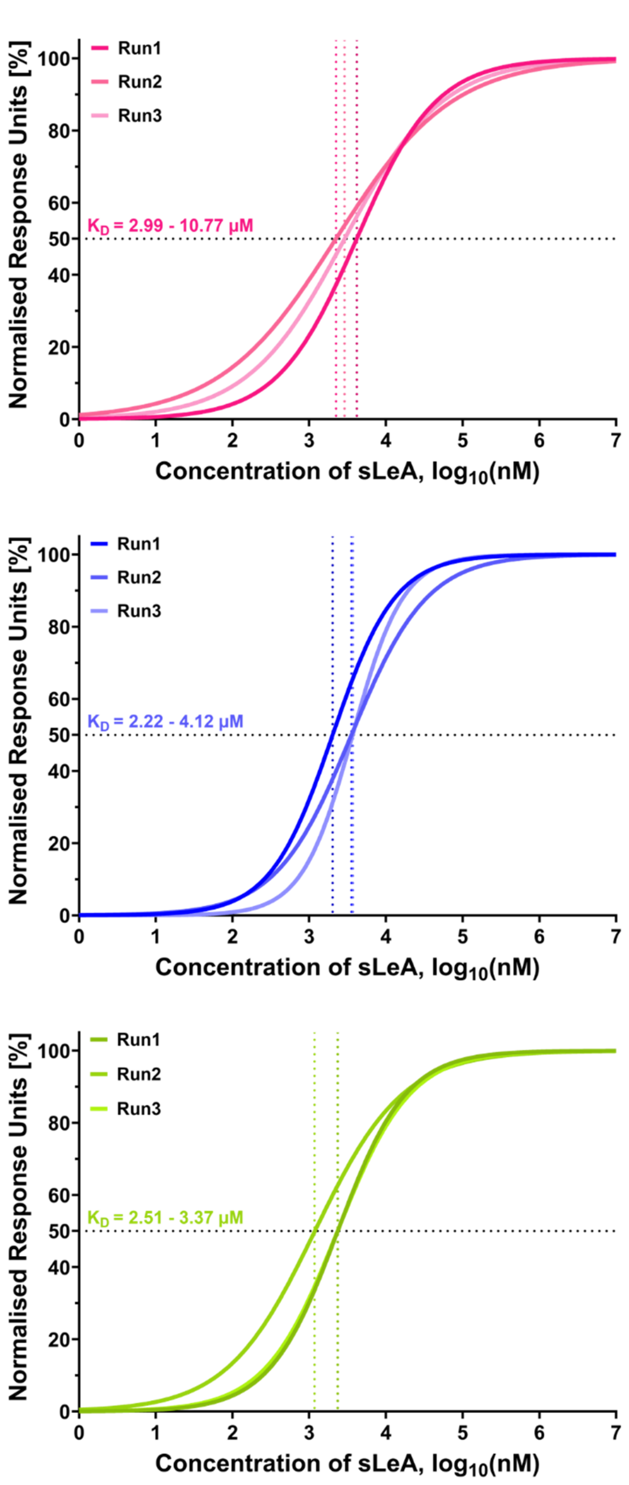

Figure S7| Fitted SPR data reveals KD ranges in micromolar range for each mAb. *SPR measurements were performed using immobilised mouse mAbs and synthetic sLeA as analyte. (**a-c**) Sensorgrams were fitted using a variable-slope, dose–response model in GraphPad Prism (v10.4.2). Three individual runs (run1–3) were performed per mAb and are shown separately. The analysis is presented for 1116-NS-19-9 in magenta (**a**), GB11 in blue (**b**), and HA8 in green (**c**).* K_D *ranges derived from the Biacore T200 evaluation software 3.2 are indicated in each panel.*

Table S1| Glycans printed on CodeLink® – glass slide. *The table lists the synthetic glycan structures that were printed on the slide with a concentration of 0.1 mM in a humidity chamber at RT. The numbering correlates with the numbering of the printing pattern found in Figure S8a as well as their SNFG representation in Figure S8e.*

No	Printed Compound	Carbohydrate Structure
**1**	Lewis A - (amino linker)	Gal(β1-3)[Fuc(α1-4)]GlcNAc(β1-1)aminopentanol
**2**	Lewis B - (amino linker)	Fuc(α1-2)Gal(β1-3)[Fuc(α1-4)]GlcNAc(β1-1)aminopentanol
**3**	Lewis X - (amino linker)	Fuc(α1-3)[Gal(β1-4)]GlcNAc(β1-1)aminopentanol
**4**	Lewis Y - (amino linker)	Fuc(α1-3)[Fuc(α1-2)Gal(β1-4)]GlcNAc(β1-1)aminopentanol
**5**	Sialyl Lewis X - (amino linker)	Fuc(α1-3)[Neu5Ac(a2-3)Gal(β1-4)]GlcNAc(β1-3)-Gal(β1-4)Glc(β1-1)aminohexanol
**6**	**Sialyl Lewis A - (amino linker)**	**Neu5Ac(α2-3)Gal(β1-3)[Fuc(α1-4)]GlcNAc(β1-1)aminopentanol**
**7**	Lactose - (amino linker)	Gal(β1-4)Glc(β1-1)aminopentanol
**8**	Sialyl Tn - (amino linker)	Neu5Ac(α2-6)GalNAc(α1-1)aminopentanol
**9**	Tn - (amino linker)	GalNAc(α1-1)
10	H-antigen type 2 - (amino linker)	Fuc(α1-2)Gal(β1-4)GlcNAc(β1-3)Gal(β1-4)Glc(β1-1)aminopentanol
**11**	Linear Hexamer - (amino linker)	Gal(β1-4)GlcN(β1-3)Gal(β1-4)GlcN(β1-3)Gal(β1-4)Glc(β1-1)aminopentanol
**12**	GM1b (cisGM1) - (amino linker)	Neu5Ac(α2-3)Gal(β1-3)GalNAc(β1-4)Gal(β1-4)Glc(β1-1)aminopentanol
**13**	GM3 - (amino linker)	Neu5Ac(α2-3)Gal(β1-4)Glc(β1-1)aminopentanol
**14**	Sialyllacto-N-tetraose - (amino linker)	Neu5Ac(α2-3)Gal(β1-3)GlcNAc(β1-3)Gal(β1-4)Glc(β1-1)aminopentanol
**15**	SLPG (S-i, nHM1) - (amino linker)	Neu5Ac(α2-3)Gal(β1-4)GlcNAc(β1-3)Gal(β1-4)GlcNAc(β1-3)Gal(β1-4)Glc(β1-1)aminopentanol
	Mouse IgG	Unrelated mouse IgG to confirm secondary binding
	CRM_{197}	Carrier protein used during immunisation

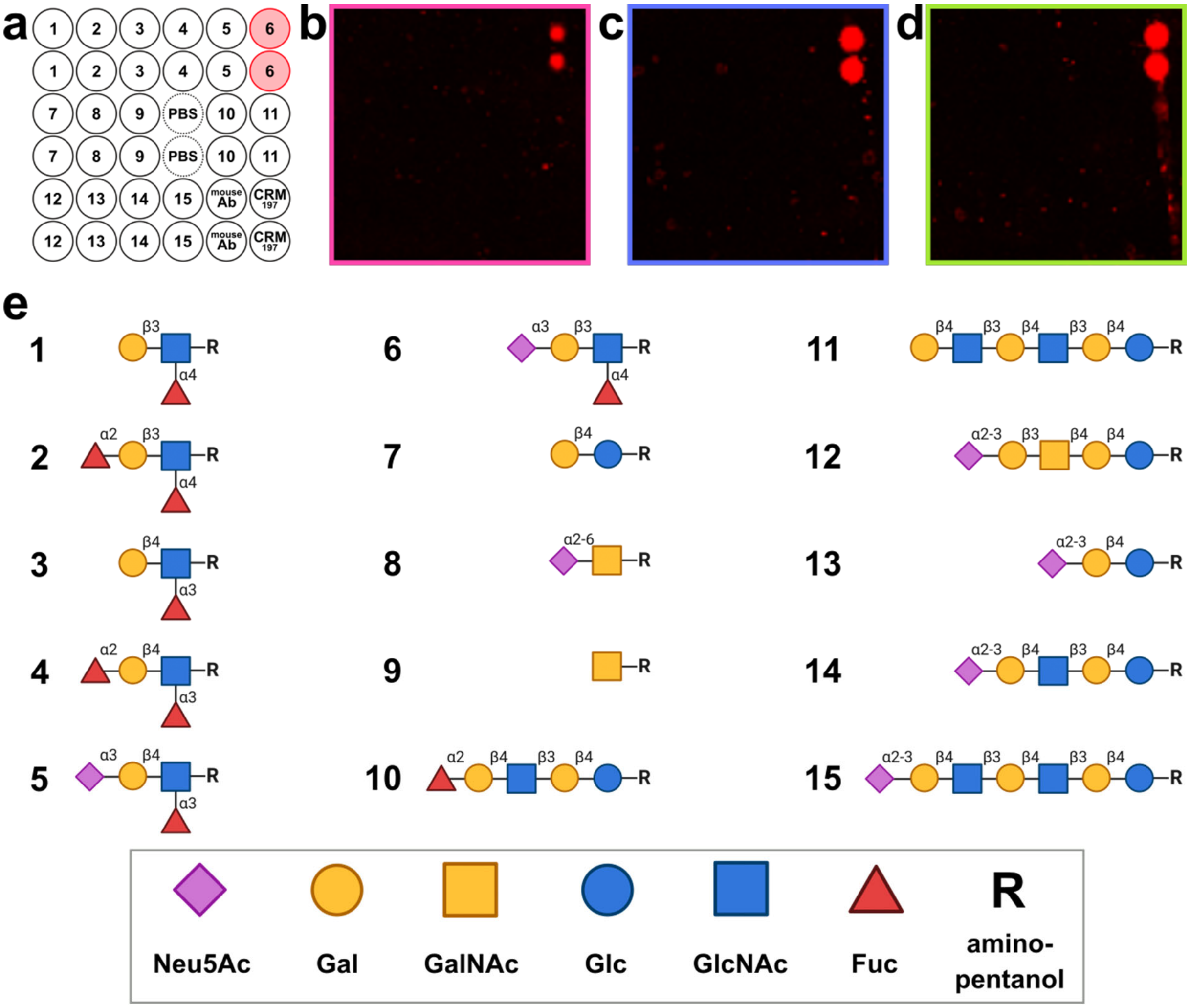

Figure S8| The mAbs show a high specificity towards sLeA only. *(**a**) Printing pattern of the glycan array. The red dots (No. 6) indicate the locations where sLeA is printed. Each number corresponds to the printed glycan structure, which are represented in the "Symbol Nomenclature for Glycans" (SNFG) as shown in (**e**). (**b-d**) Depict one out of four repetitions of the glycan array, demonstrating that the mAbs 1116-NS-19-9 (**b**), GB11 (**c**), and HA8 (**d**) exhibit high antigen specificity, binding exclusively to sLeA. The SNFG representations were created with BioRender.com.*

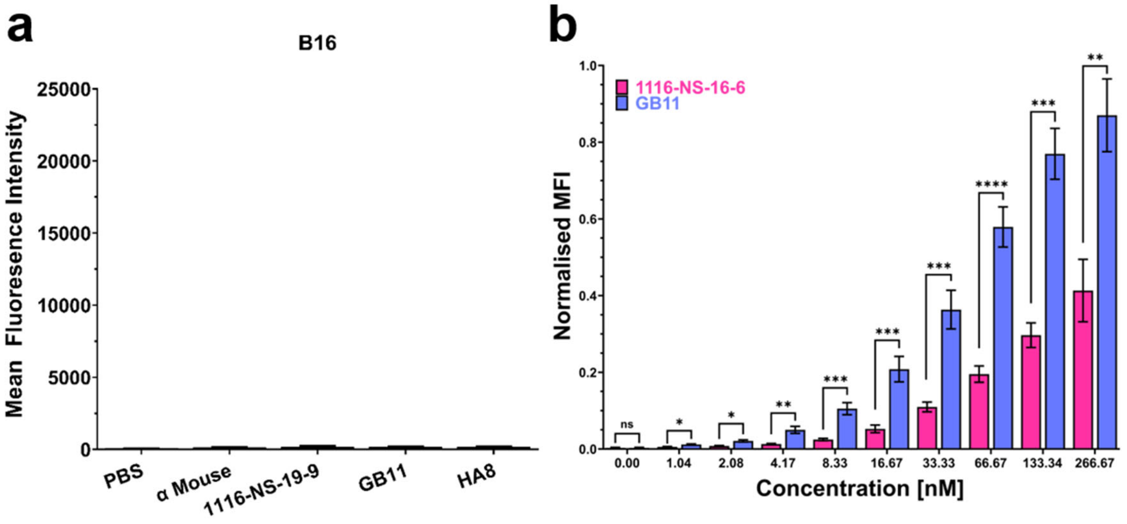

Figure S9| GB11 binds significantly stronger to sLeA expressing mouse melanoma cells. *(**a**) Mean fluorescence intensity (MFI) with standard error of the mean (SEM) for 1116-NS-19-9 (magenta), GB11 (blue), and HA8 (green) on B16 mouse melanoma cells. Five independent assays were conducted for each sample, using 5 µg/mL of the respective mAbs. Statistical analysis was performed using One-Way ANOVA. (**b**) Titration binding assay performed on B16-FUT3+ cells for GB11 (blue) and 1116-NS-19-9 (magenta). The MFI was normalised to the highest measured MFI for each mAb. Error bars represent the SEM. PBS and secondary-only samples are omitted for clarity. Statistical analysis was conducted using One-Way ANOVA (ns = not significant, *p ≤ 0.05, **p ≤ 0.01, ***p ≤ 0.001, ****p ≤ 0.0001).*

Table S2| X-ray data collection and model refinement statistics. *Structures for apo (PDB ID: 9I6Q) and holo (PDB ID: 9I9H) forms were obtained. Values in parentheses refer to the highest resolution shell.*

	GB11 apo	*GB11 sLeA*
Wavelength	0.9184	0.9184
Reflections	469197	122337
Unique	40412	40947
Space group	P4	R1
Cell dimensions		
a, b, c	109.160, 109.160, 40.268	39.838, 108.697, 108.920
α, β, γ	90.00, 90.00, 90.00	89.60, 87.34, 89.25
Resolution	54.58-1.86 (1.90-1.86)	26.98-2.90 (3.02-2.80)
Completeness	100 (99.6)	98.2 (93.4)
I/σ(I)	7.7 (0.9)	2.6 (0.6)
CC1/2	0.996 (0.376)	0.915 (0.189)
Rmerge	0.202 (2.871)	0.327 (2.035)
Multiplicity	11.6 (11.6)	3 (3.2)
Refinement		
Resolution	48.82-1.68	26.70-2.96
Rwork/Rfree	0.205/0.240	0.210/0.248
Number of monomers	1	4
Average B factors	23.74	62.86
GB11	22.879	63.1
Ligand	-	78.83
Solvent	37.523	52.2
RMS Z-score		
Bonds	0.758	0.441
Angles	0.973	0.759
Ramachandran		
Favoured	413 (97.18%)	1588 (93.41%)
Allowed	11 (2.59%)	100 (5.87%)
Disfavoured	1 (0.24%)	15 (0.88%)

Table S3| Glycosidic angles of sLeA in the bound state.

	NeuNAca(2–3)Gal	Galβ(1–3)GlcNAc	Fucα(1–4) GlcNAc
	Φ[°], Ψ[°]	Φ[°], Ψ[°]	Φ[°], Ψ[°]
sLeA, bound to 1116-NS-19-9<br>PDB-ID: 6XTG	(49.5, 122.7)	(-82.0, 128.3)	(-74.2, -96.8)
sLeA, bound to GB11<br>PDB-ID: 9I9H	(48.1, 131.8)	(-84.2, 115.9)	(-83.3, -106.3)

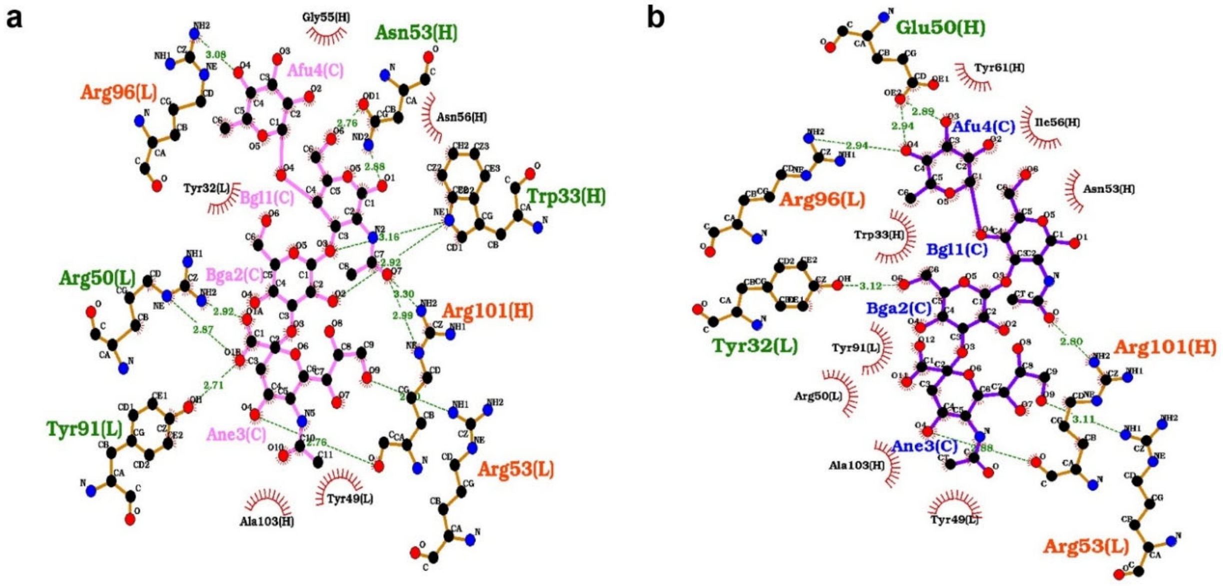

Figure S10| Ligplot results of the minimised docked complex of 1116-NS-19-9 and GB11 from AutoDock Vina. *(**a**) The plot represents the h-bond distance and interacting residues of the complex 1116-NS-19-9 with sLeA and in (**b**) for the complex of GB11 with sLeA. The binding sites residues forming h-bond interaction in both the complexes are named in orange-red colour and residues that are specific for each of the mAbs are named in olive green.*

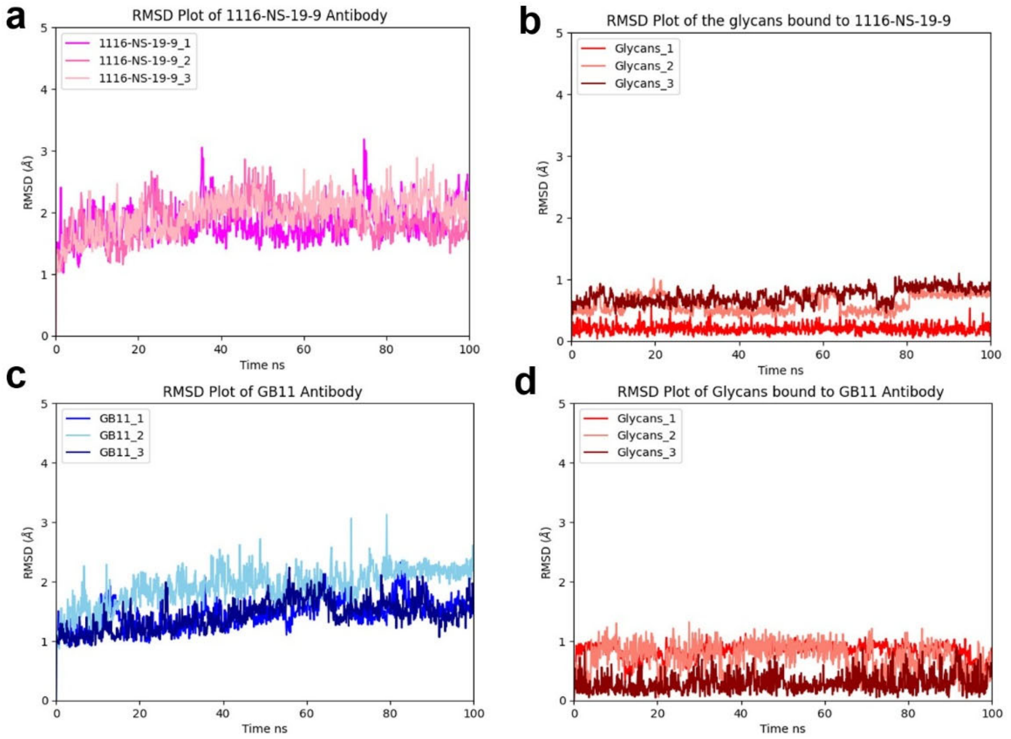

1116-NS-19-9_Complex	Average RMSD
1116-NS-19-9_replicate_1	1.891 ± 0.324
1116-NS-19-9_replicate_2	1.703 ± 0.485
1116-NS-19-9_replicate_3	1.890 ± 0.319
Glycans_1	0.197 ± 0.306
Glycans_2	0.598 ± 0.374
Glycans_3	0.725 ± 0.291

GB11_Complex	Average RMSD
GB11_replicate_1	1.447 ± 0.266
GB11_replicate_2	1.920 ± 0.272
GB11_replicate_3	1.425 ± 0.287
Glycans_replicate_1	0.830 ± 0.307
Glycans_replicate_2	0.726 ± 0.235
Glycans_replicate_3	0.295 ± 0.256

Figure S11| SLeA binding stabilises GB11 and 1116-NS-19-9 complexes. *(**a**) The last 100 ns averaged backbone atom RMSD values of all three replicates of 1116-NS19-9 exhibit significant overlap, indicating consistency across the replicates. (**b**) The averaged backbone atom RMSD of the tetrasaccharide bound to 1116-NS19-9 remained consistently lower than that of 1116-NS19-9, suggesting a stable retention of sLeA within the binding pocket. (**c**) The last 100 ns averaged backbone atom RMSD values of all three replicates of GB11 show good overlap within the admissible range of 3.0 Å.(**d**) sLeA bound to GB11 exhibited a backbone atom deviation with an RMSD value of less than 1.0 Å across all three replicates, indicating stable ligand binding.*

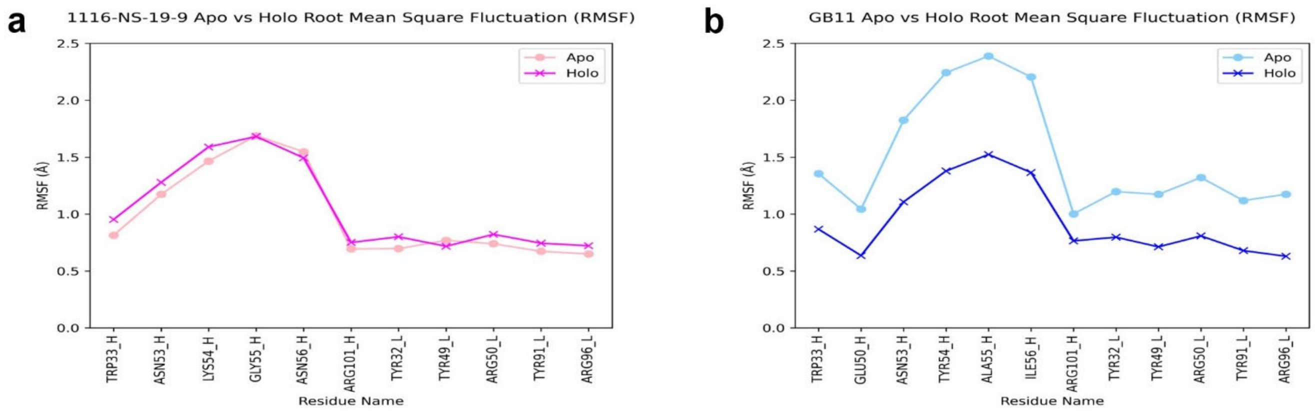

Figure S12| Tetrasaccharide binding reduces binding site flexibility in GB11 but not in 1116-NS-19-9. *The RMSF values were computed and averaged across replicates, focusing on binding site residues of 1116-NS-19-9 and GB11. (**a**) 1116-NS19-9 exhibited minimal fluctuations in the RMSF values of the binding site residues in both the apo and holo forms. (**b**) In contrast, GB11 when comparing its apo and holo forms, showed reduced fluctuations in the binding site residues in the holo form upon tetrasaccharide binding, suggesting increased backbone rigidity.*

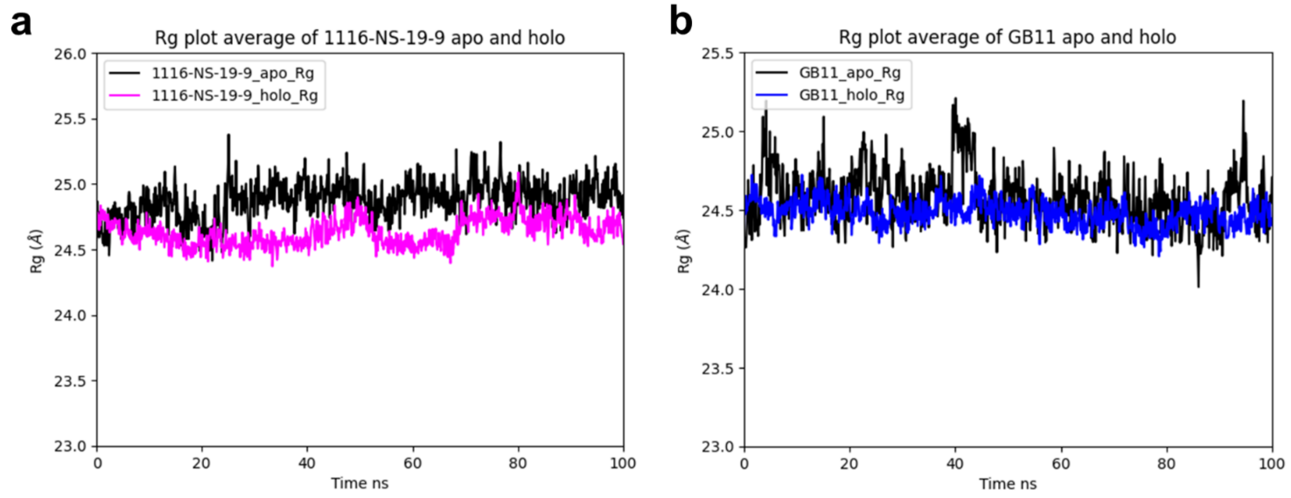

Figure S13| The average radius of gyration (Rg) apo and holo mAb. *The plot reveals that the protein structure maintains a high degree of compactness, with a Rg value consistently fluctuating within the acceptable range of < 1 Å for both 1116-NS-19-9 (**a**) and GB11 (**b**) in both their apo and holo forms. However, in the GB11 holo form the compactness looks well preserved.*

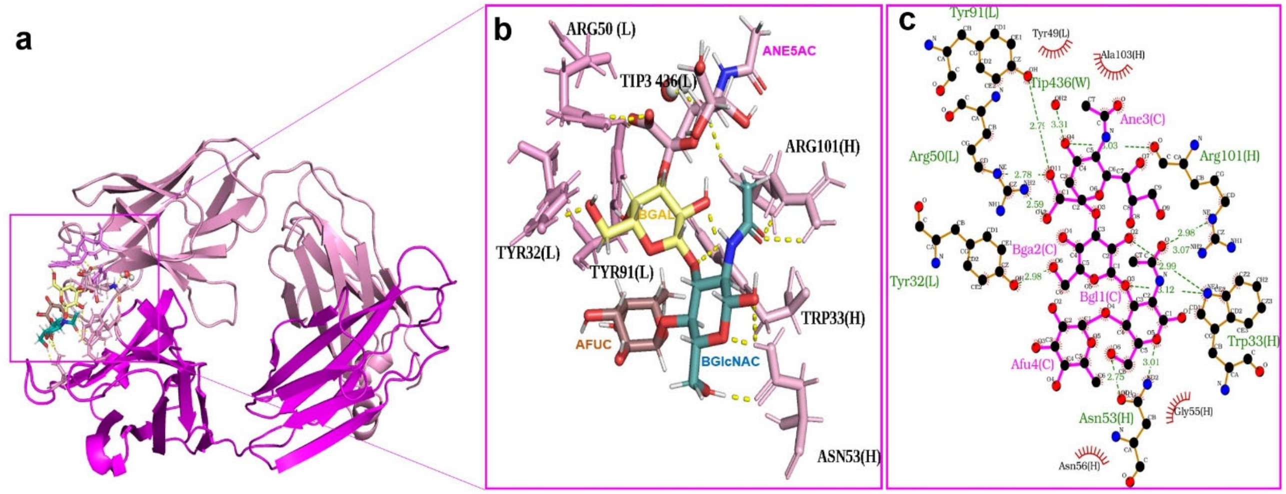

Figure S14| 1116-NS-19-9 binding site for sLeA with lowest interaction energy score. *(**a**) Binding site of the 1116-NS-19-9 mAb. (**b**) Displaying the residues engaged in forming polar H-bond contacts with the glycan residues (**c**) Its 2D Ligplot showing the H-bond distance between the glycans and the key residues.*

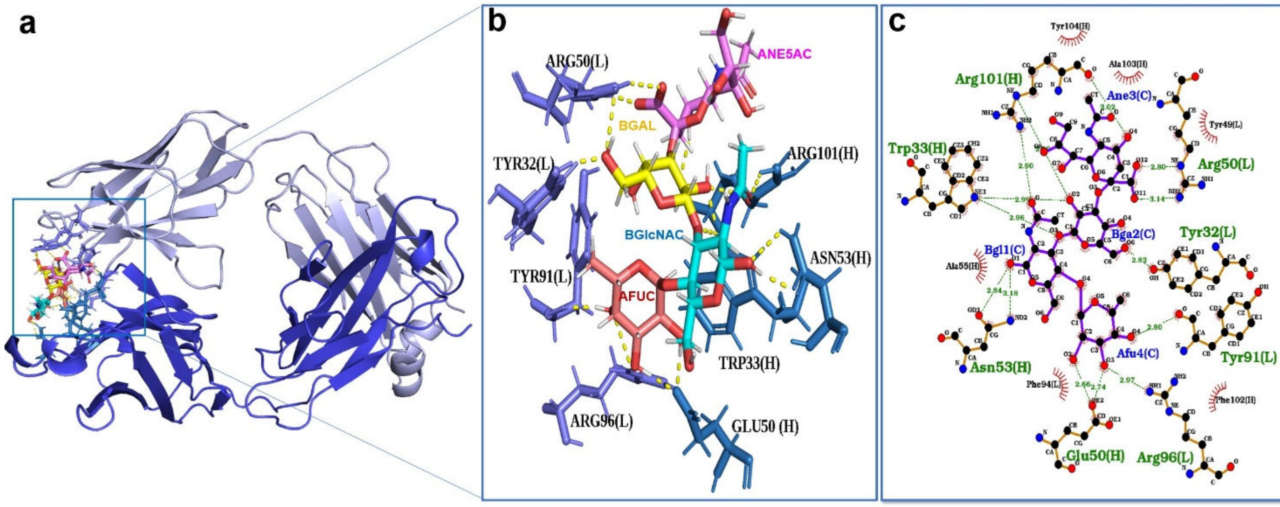

Figure S15| GB11 binding site for sLeA with lowest interaction energy score. *(**a**) Binding site of the GB11 mAb. (**b**) It reveals the vital residues involved in forming polar H-bond contacts with the glycan residues. (**c**) A 2D Ligplot provides a visualization of the H-bond distances between the glycans and the critical residues.*

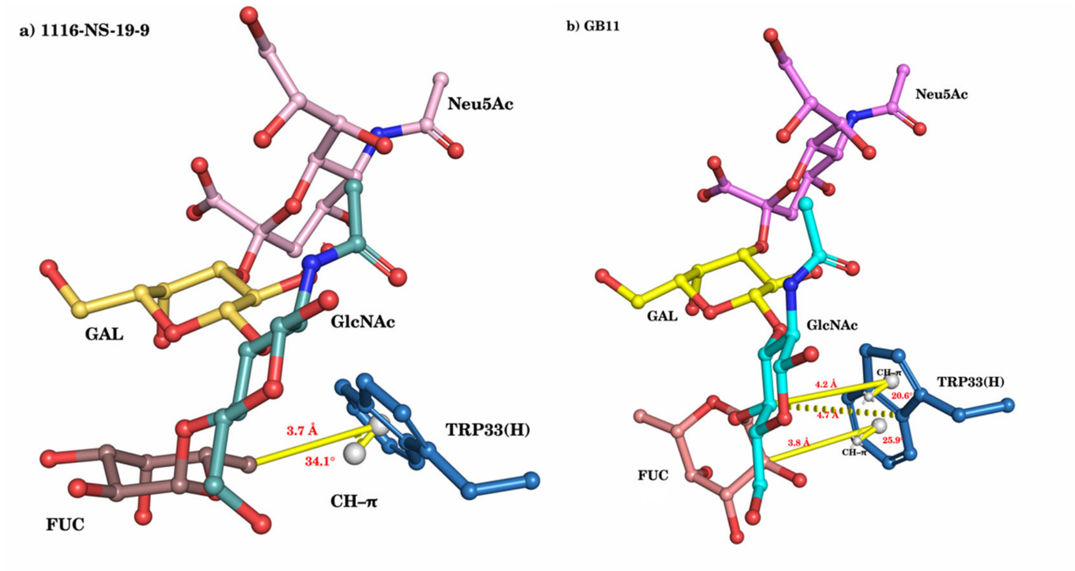

Figure S16| *CH–π geometry of TRP33(H)–glycan contacts in representative low-energy conformations. (**a**) 1116-NS-19-9 shows a single TRP33 (H)–Fuc interaction (Fuc: C6–π centroid distance 3.7 Å; ring-normal angle 34.1°). (**b**) GB11 shows additional TRP33(H) CH–π contacts including BGL C4 (4.2 Å; 20.6°) and FUC C2 (3.8 Å; 25.9°), with BGL C4 also forming close edge contacts to TRP33 CD2 (4.7 Å). Plots were generated with Arpeggio web server [36].*

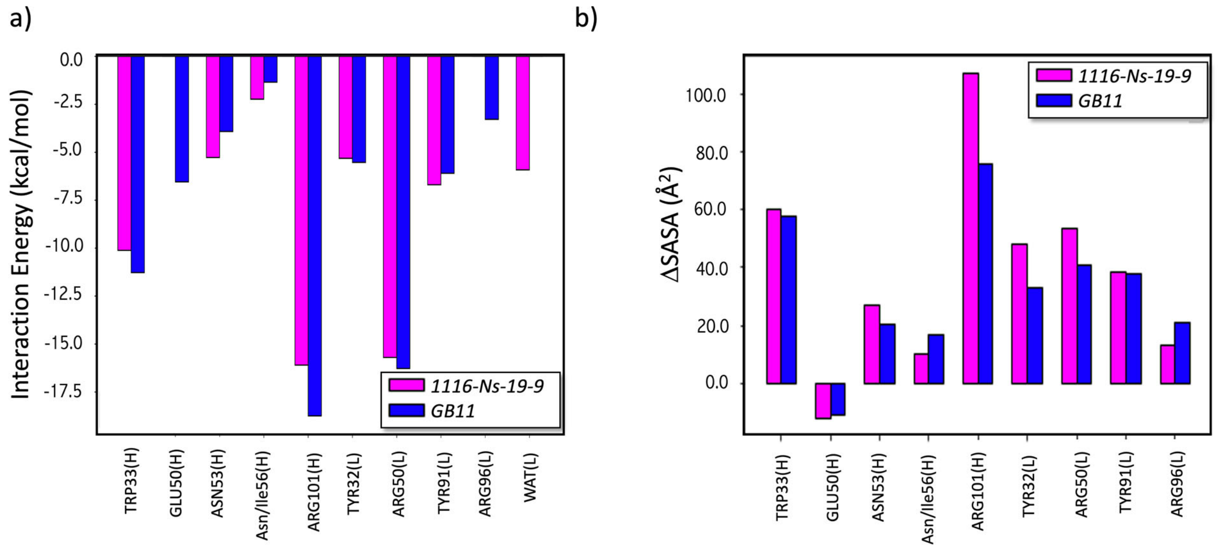

Figure S17| *(**a**) Average total interaction energy (electrostatic + van der Waals) of binding-site residues from the heavy and light chains of 1116-NS-19-9 (pink) and GB11 (blue), calculated over the final 100 ns of the MD simulations. (**b**) Difference in the average solvent-accessible surface area (ΔSASA) of residues within the ligand-binding pocket between the holo and apo states, evaluated over the final 100 ns of the three independent MD simulations. The ΔSASA analysis shows that sLeA binding markedly reduces the solvent exposure of and Trp33(H), Arg101(H) and Arg50(L), consistent with their strong interaction energies. Notable differences in ΔSASA between 1116-NS-19-9 and GB11 are observed for Arg101(H), Tyr32(L0) and Arg50(L) suggesting that multiple site mutations in 1116-NS-19-9 modulate sLeA interactions with the protein matrix. The N56I mutation induces a minor increase of the SASA of residues 56 and 96. These differences are however within the uncertainties of these calculations (see Table S4).*

Table S4| *Solvent-accessible surface areas (SASA) of selected side chains in the 1116-NS-19-9 and GB11 mAbs, calculated using the sasa plugin in VMD [37]. SASA values were averaged over the final 100 ns of three independent MD trajectories for both apo and holo systems using a probe radius of 1.4 Å. ΔSASA was defined as the difference between the average SASA of each residue in the holo and apo states. The difference in ΔSASA upon ligand binding between 1116-NS-19-9 and GB11 (ΔΔSASA) is reported in the final column. Standard deviations and propagated uncertainties for ΔSASA and ΔΔSASA were calculated using error propagation. SASA values are given in Å².*

	**1116-NS-19-9**				**GB11**				
	SASA(apo)	SASA(holo)	ΔSASA		SASA(apo)	SASA(holo)	ΔSASA		Δ(ΔSASA)
Trp33(H)	71.1±22.5	10.9±4.1	60.2±22.9		65.4±10.3	7.7±4.1	57.8±11.1		-2.4±25.4
Glu50(H)	18.2±9.3	30.0±8.8	-11.9±12.8		14.1±9.7	25.0±13.9	-10.9±17.0		1.0±21.3
Asn53(H)	29.3±8.9	2.3±2.1	27.0±9.1		25.9±4.8	5.5±6.6	20.4±8.2		-6.6±12.3
**Asn/Ile56(H)**	**70.4±11.1**	**60.3±8.8**	**10.2±14.1**		**86.5±8.0**	**69.7±15.0**	**16.8±17.0**		**6.6±22.1**
**Thr/Val100(H)**	**10.0±4.6**	**2.5±2.2**	**7.5±5.1**		**17.3±5.0**	**2.1±2.0**	**15.2±5.4**		**7.7±7.4**
Arg101(H)	141.3±26.0	34.2±8.8	107.1±27.3		108.0±11.9	32.2±8.7	75.8±14.8		-31.4±31.1
Phe102(H)	18.5±17.4	4.0±2.3	14.5±17.5		15.7±7.2	6.8±5.9	8.9±9.3		-5.6±19.9
Tyr104(H)	91.8±19.4	74.3±16.2	17.5±25.3		101.8±18.4	69.1±14.0	32.7±23.1		5.2±34.3
Tyr32(L)	95.4±14.3	47.3±10.3	48.0±17.6		81.4±10.9	48.7±12.0	32.7±16.2		-15.3±23.9
Tyr49(L)	82.5±19.4	31.2±7.4	51.3±20.8		79.9±13.1	33.9±8.8	46.0±15.7		-5.2±26.3
Arg50(L)	117.3±19.6	63.7±14.5	53.6±24.4		109.1±21.2	68.3±16.4	40.8±26.8		-12.9±36.3
Tyr91(L)	46.6±12.7	8.1±4.8	38.5±13.5		42.9±12.5	5.1±4.7	37.8±13.4		-0.7±19.0
Asp92(L)	58.7±8.9	53.3±9.9	5.4±13.3		49.2±8.8	54.3±9.5	-5.1±12.9		-10.5±18.5
Phe94(L)	91.6±21.0	120.6±21.9	-29.0±30.3		93.1±22.2	112.2±30.9	-19.1±38.0		9.9±48.6
Arg96(L)	33.9±9.4	20.8±6.8	13.0±11.5		38.4±12.6	17.6±8.9	20.8±15.4		7.8±19.2
sLeA		397.1±24.2				404.4±31.8			

Table S5| Chemical Shift Assignment of sialyl Lewis A. *The table contains the chemical shift values in ppm for ^{1}H and ^{13}C atoms of sLeA. The commercially available sLeA comprises a mixture of α- and β-GlcNAc anomers, attributable to the presence of a free OH-group at the CH1 of GlcNAc. Consequently, resonances corresponding to both α- and β-GlcNAc are observed, and are represented with α- and β- respectively.*

	Position	^{1}H [ppm]	^{13}C [ppm]
α-GlcNAc	1	5.05	91.0
	2	4.07	54.0
	3	4.10	74.2
	4	3.68	72.4
	5	3.91	71.4
	6	3.80	59.7
	NAc-Methyl	1.97	22.2
β-GlcNAc	1	4.65	94.8
	2	3.79	56.8
	3	3.99	76.1
	4	3.66	72.3
	5	3.49	75.5
	6	3.88	59.7
	NAc-Methyl	1.97	22.2
Gal-α-GlcNAc	1	4.49	102.8
Gal-β-GlcNAc	1	4.48	102.8
Gal	2	3.43	68.8
	3	3.98	75.6
	4	3.83	66.9
	5	3.46	74.6
	6	3.62	61.5
Fuc	1	4.94	98.0
	2	3.72	67.8
	3	3.81	69.1
	4	3.71	72.0
	5	4.81	66.8
	6	1.10	15.3
Neu5Ac	3-1	2.70	40.0
	3-2	1.69	40.0
	4	3.61	68.5
	5	3.78	51.7
	6	3.55	72.7
	7	3.54	68.0
	8	3.78	71.8
	9-1	3.75	62.2
	9-2	3.58	62.2
	NAc-Methyl	1.96	22.1

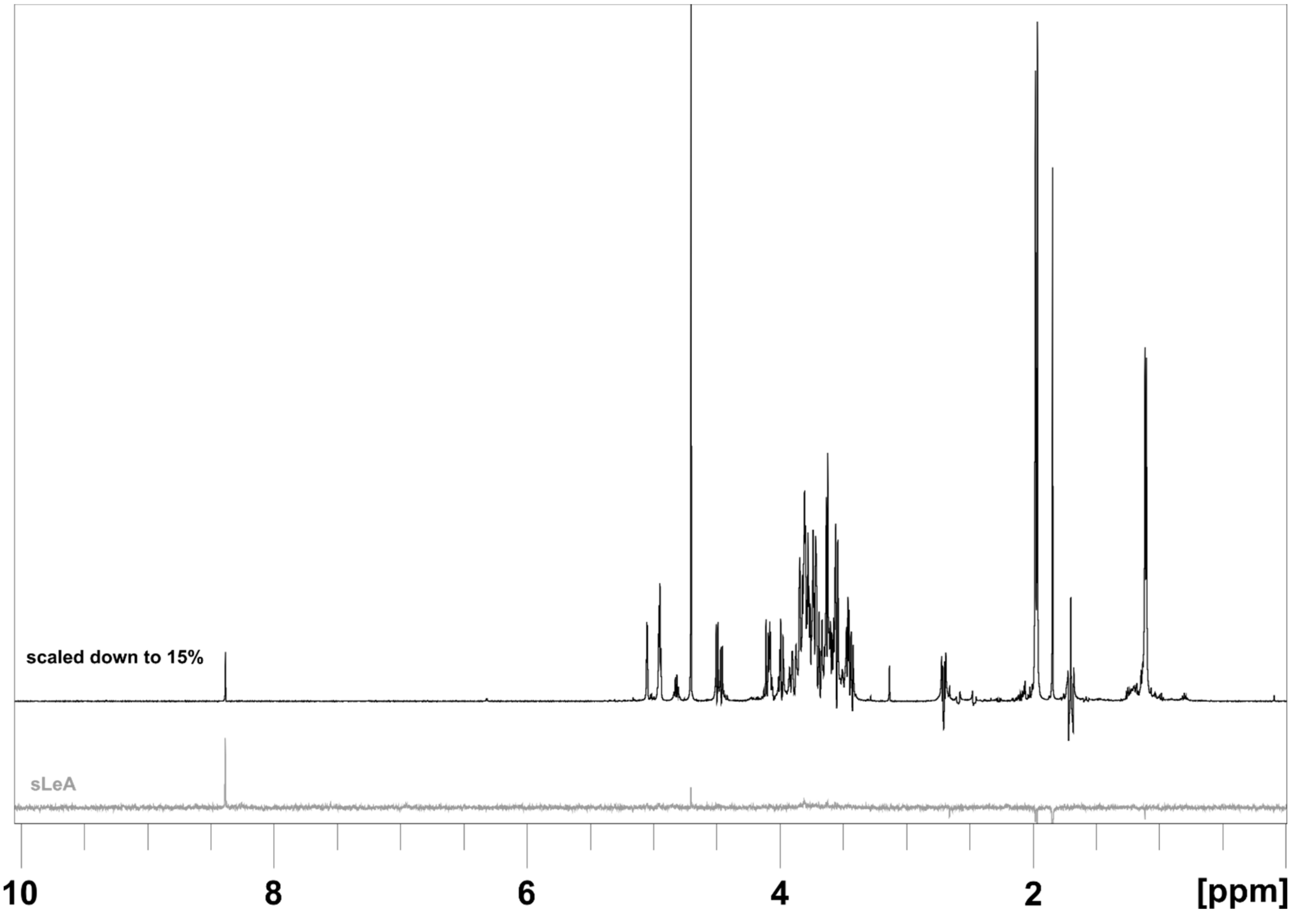

Figure S18| In the absence of a protein, the STD spectrum of sLeA exhibits minimal saturation. *Protein saturation was achieved using low-power Gaussian-shaped pulses at 8.25 ppm, with a total duration of 2 seconds. The grey spectrum illustrates the STD spectrum of sLeA in the absence of a protein.*

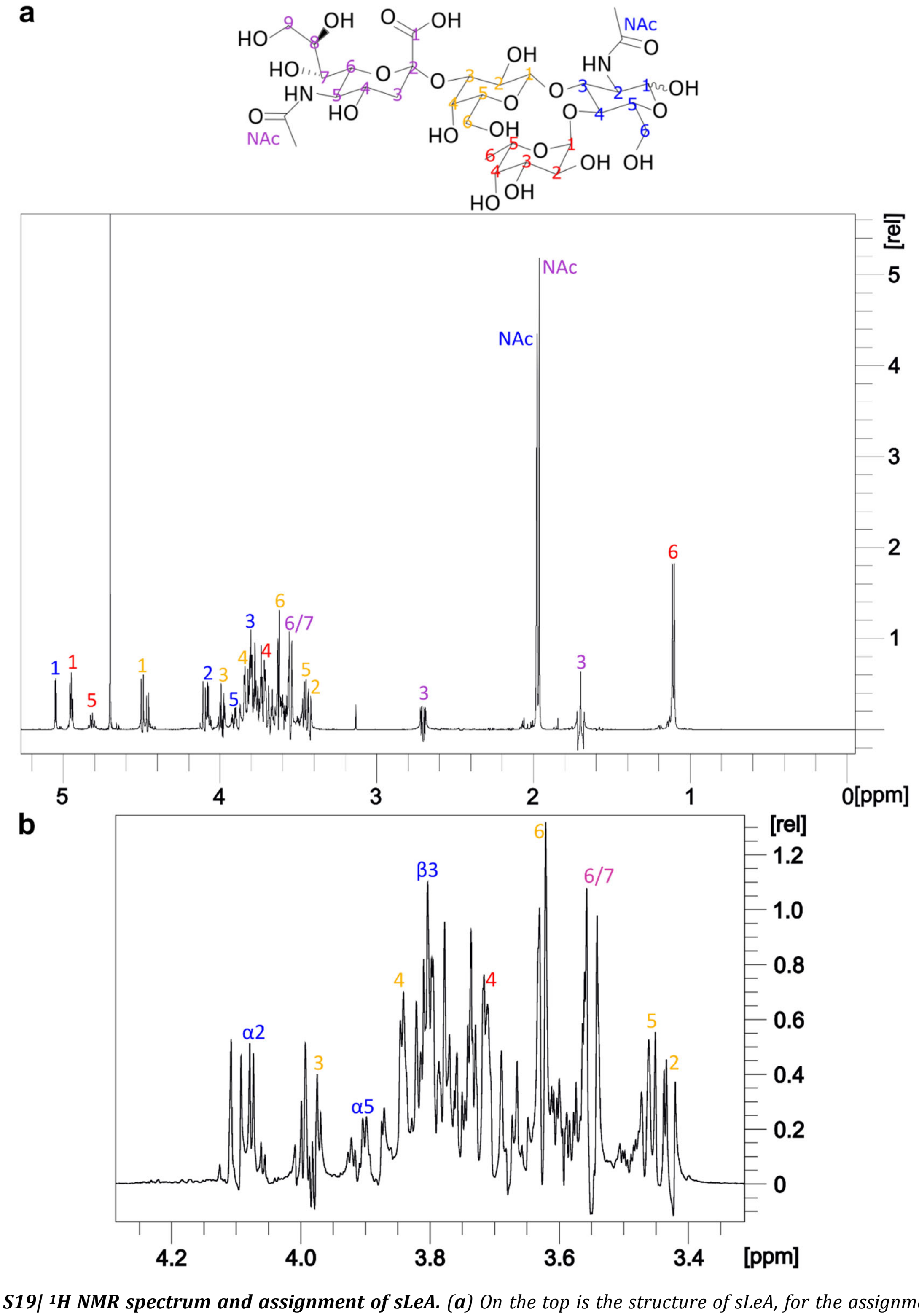

Figure S19| ¹H NMR spectrum and assignment of sLeA. *(**a**) On the top is the structure of sLeA, for the assignment each carbon atom is represented with numbers in the colours of the monosaccharides in the SNFG representation, GlcNAc (blue), Fuc (red), Gal (yellow) and Neu5Ac (purple). These numbers are used on the respective resonances of sLeA in the ¹H-spectrum. (**b**) The zoomed-in ¹H spectrum spans the range between 4.2 and 3.4 ppm. Anomers of α- and β-GlcNAc are represented with α and β respectively. The assignment presented herein is relevant to the STD NMR analysis.*

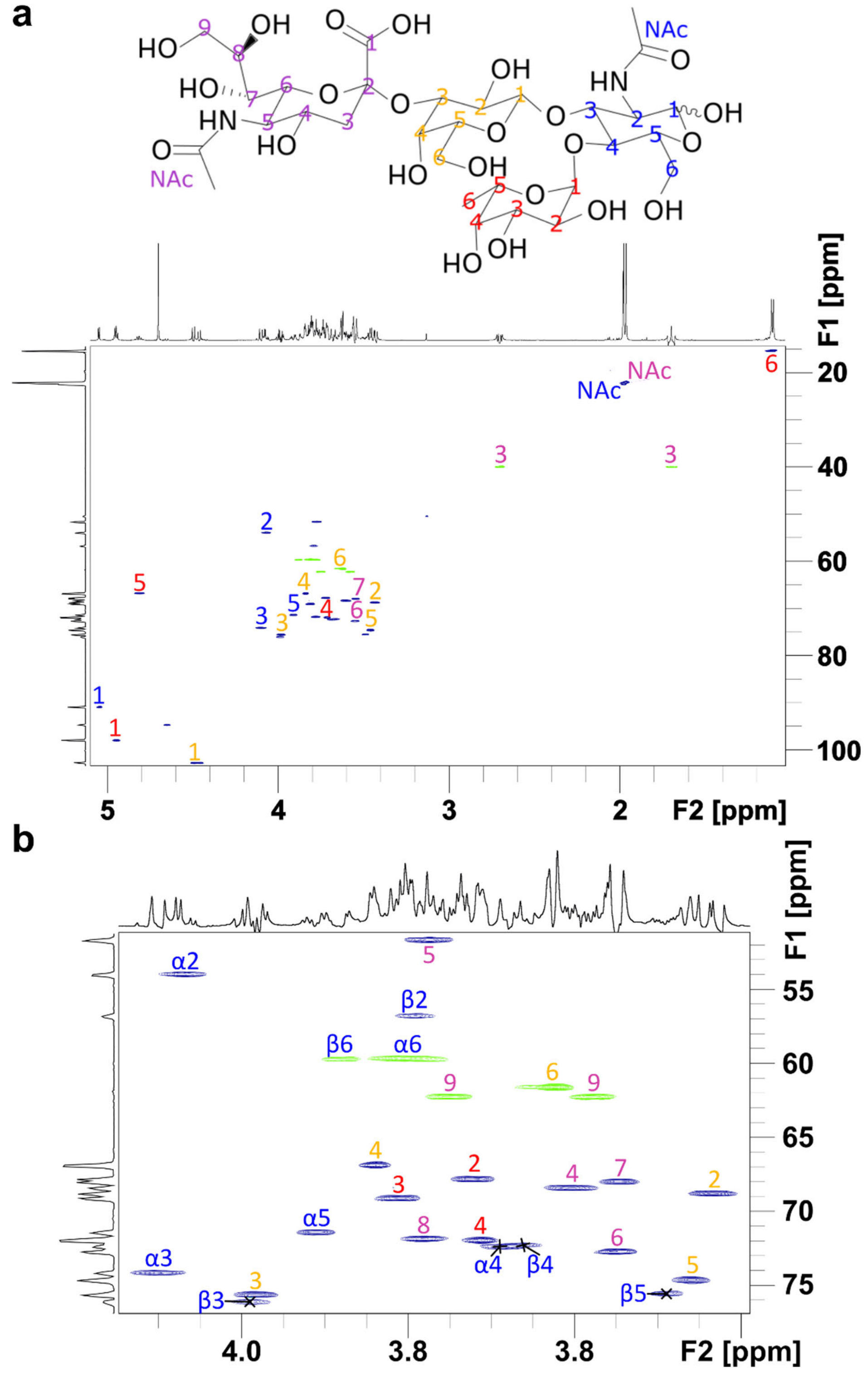

Figure S20| ^{1}H-^{13}C HSQC and assignment of sLeA. *(**a**) On the top is the structure of sLeA. For the assignment each carbon atom is represented with numbers in the colours of the monosaccharides in the SNFG representation, GlcNAc (blue), Fuc (red), Gal (yellow) and Neu5Ac (purple). These numbers are used on the respective resonances of sLeA in the ^{1}H-^{13}C HSQC. (**b**) ^{1}H-^{13}C HSQC zoomed in between 55 and 75 ppm for F1 (^{13}C) and 4.2 and 3.6 ppm for F2 (^{1}H). Anomers of α- and β-GlcNAc are represented with α and β respectively. In (**a**) we present the assignment used for the STD NMR analysis and in (**b**) respective assignment for α- and β-GlcNAc.*

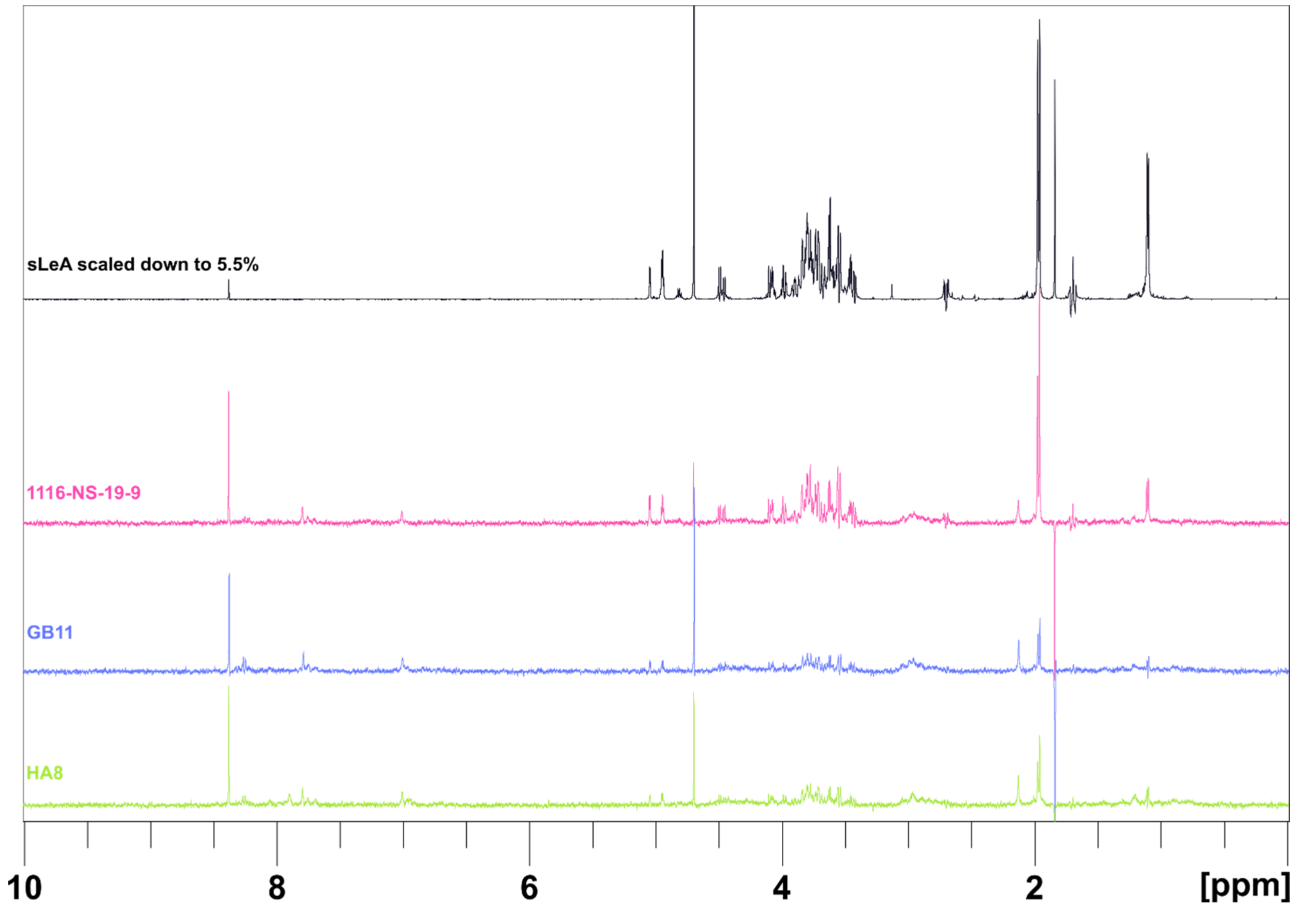

Figure S21| The STD spectra of sLeA demonstrate interaction with all three mAbs. *At the top, the ^{1}H reference spectrum of 200 µM sLeA, reduced to 5.5%, is depicted in black. In the lower section of the image, the STD spectra of 200 µM sLeA are presented, recorded concurrently with 1116-NS-19-9 (magenta), GB11 (blue), or HA8 (green) at a protein concentration of 6.7 µM each. It is evident that all three monoclonal antibodies bind to sLeA. Furthermore, 1116-NS-19-9 exhibits on average higher STD effects, suggesting differences in binding kinetics compared to GB11 and HA8.*

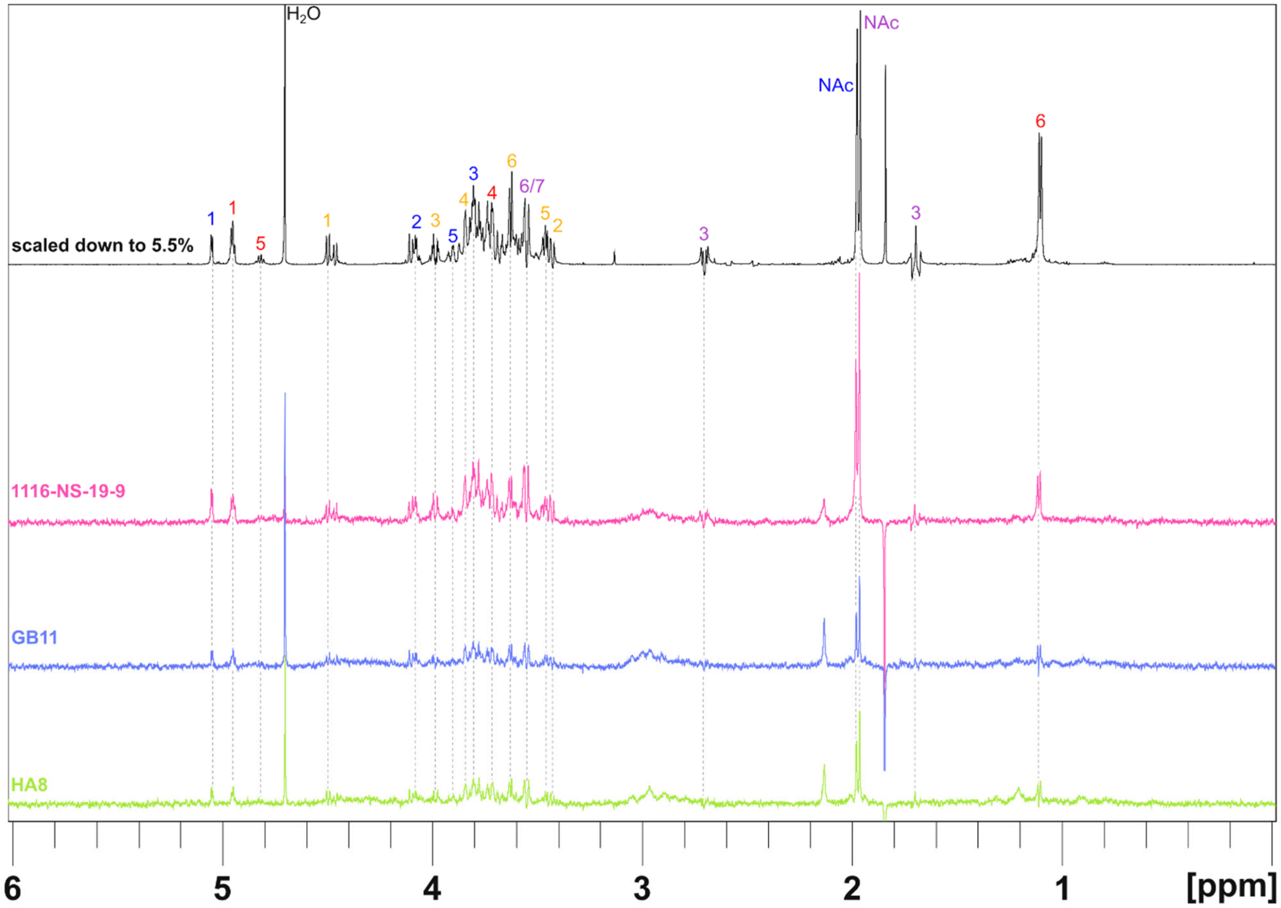

Figure S22| The STD spectra depict the resonances involved in the interaction between sLeA and the respective antibody*. The ^{1}H reference spectrum of 200 µM sLeA, reduced to 5.5%, is illustrated in black at the top. Each hydrogen atom utilized for STD effect measurement is labelled with numbers corresponding to the monosaccharide colours in the SNFG representation: GlcNAc (blue), Fuc (red), Gal (yellow), and Neu5Ac (purple). In the lower section of the image, the STD spectra of 200 µM sLeA are displayed, recorded in conjunction with 1116-NS-19-9 (magenta), GB11 (blue), and HA8 (green) at a protein concentration of 6.7 µM each.*

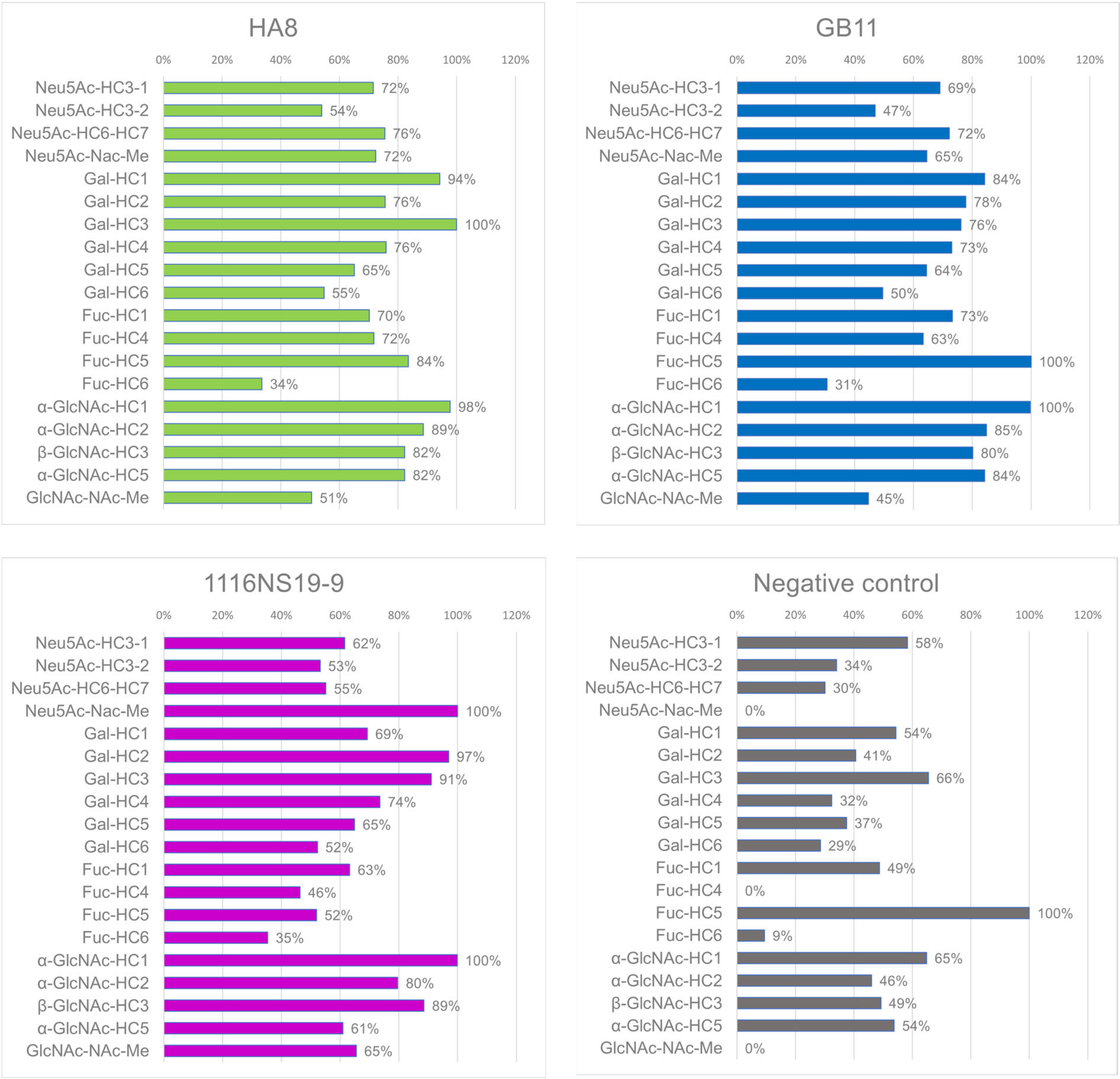

Figure S23| *STD NMR effects on sLeA glycan in the presence of GB11, HA8 and 1116-NS-19-9. The values represent absolute STD intensities referenced to the most intense STD effect, which is expressed as 100%. The values in the negative control originate from the sample without any antibody present to account for any unspecific direct saturation of sLeA glycan.*

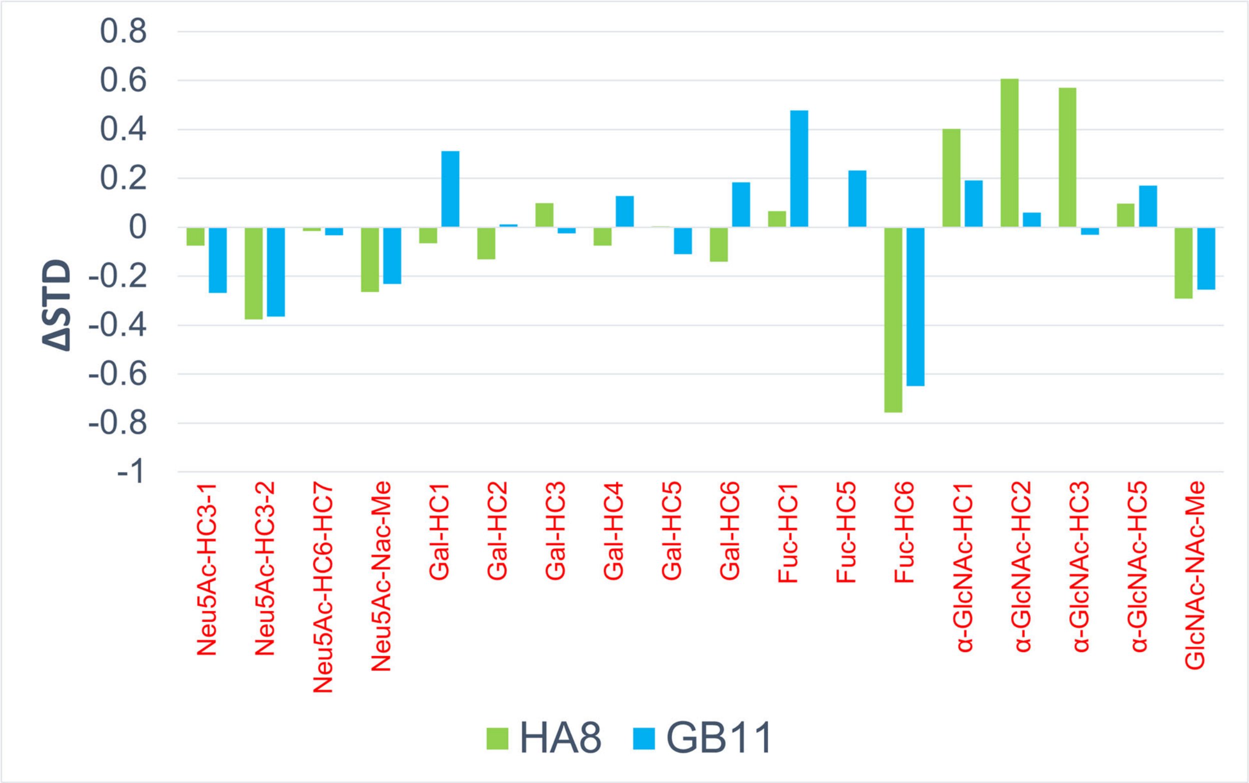

Figure S24| *DEEP-STD NMR factors (ΔSTD) for sLeA in the presence of HA8 and GB11 antibodies. Positive ΔSTD values indicate stronger saturation during irradiation at 6.69 ppm and negative values – during irradiation at -0.74 ppm.*

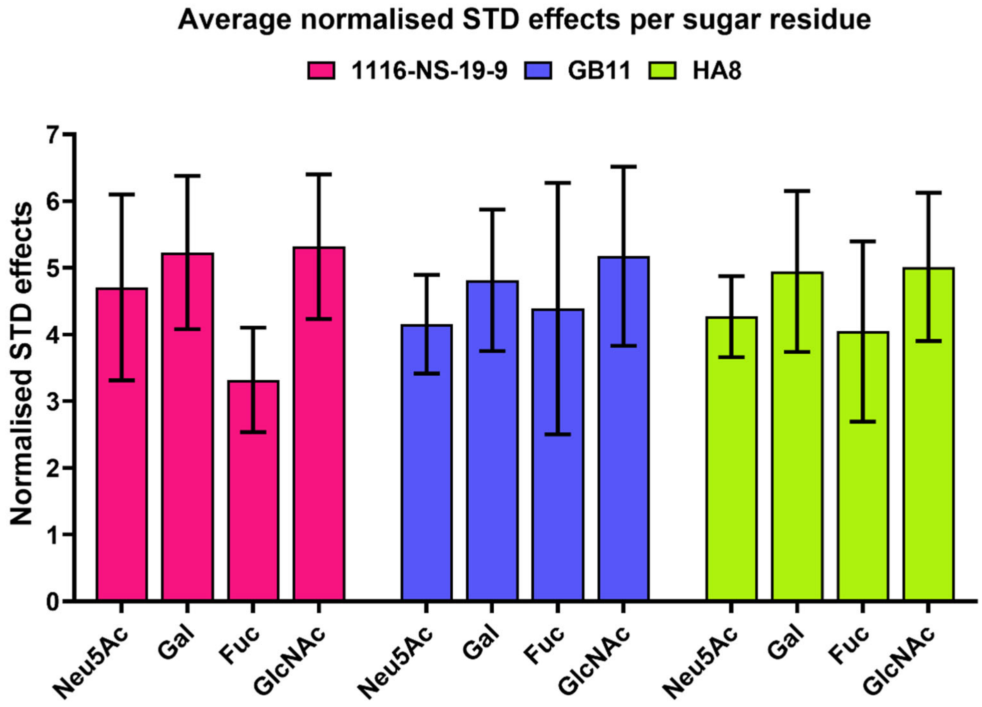

Figure S25| *GB11 and HA8 show more uniform STD NMR saturation across sLeA glycan units than 1116-NS-19-9. Average normalised STD effects per sugar residue are shown for each antibody: 1116-NS-19-9 in magenta, GB11 in blue, and HA8 in green. GB11 and HA8 display more uniform saturation across all four glycan units of sLeA, whereas 1116-NS-19-9 shows notably weaker saturation at the fucose residue, indicating less engagement of this part of the epitope.*

REFERENCES

(1) Broecker, F.; Hanske, J.; Martin, C. E.; Baek, J. Y.; Wahlbrink, A.; Wojcik, F.; Hartmann, L.; Rademacher, C.; Anish, C.; Seeberger, P. H. Multivalent Display of Minimal Clostridium Difficile Glycan Epitopes Mimics Antigenic Properties of Larger Glycans. *Nat. Comm. 2016 7:1* **2016**, *7* (1), 1–12.

(2) Wu, X.; Ling, C. C.; Bundle, D. R. A New Homobifunctional P-Nitro Phenyl Ester Coupling Reagent for the Preparation of Neoglycoproteins. *Org. Lett.* **2004**, *6* (24), 4407–4410.

(3) Broecker, F.; Anish, C.; Seeberger, P. H. Generation of Monoclonal Antibodies against Defined Oligosaccharide Antigens. *Meth. Mol. Biol.* **2015**, *1331*, 57–80.

(4) Meyer, L.; López, T.; Espinosa, R.; Arias, C. F.; Vollmers, C.; DuBois, R. M. A Simplified Workflow for Monoclonal Antibody Sequencing. *PLoS One* **2019**, *14* (6).

(5) Motohashi, K. A Novel Series of High-Efficiency Vectors for TA Cloning and Blunt-End Cloning of PCR Products. *Sci. Rep. 2019 9:1* **2019**, *9* (1), 1–11.

(6) Khilji, S. K.; Goerdeler, F.; Frensemeier, K.; Warschkau, D.; Lühle, J.; Fandi, Z.; Schirmeister, F.; Chen, Z. A.; Turak, O.; Mallagaray, A.; Boerno, S.; Timmermann, B.; Rappsilber, J.; Seeberger, P. H.; Moscovitz, O. Generation of Glycan-Specific Nanobodies. *Cell Chem. Biol.* **2022**, *29* (8), 1353-1361.e6.

(7) Disney, M. D.; Seeberger, P. H. The Use of Carbohydrate Microarrays to Study Carbohydrate-Cell Interactions and to Detect Pathogens. *Chem. Biol.* **2004**, *11* (12), 1701–1707.

(8) Weitzenfeld, P.; Bournazos, S.; Ravetch, J. V. Antibodies Targeting Sialyl Lewis A Mediate Tumor Clearance through Distinct Effector Pathways. *J. Clin. Invest.* **2019**, *129* (9), 3952–3962.

(9) Beilsten-Edmands, J.; Winter, G.; Gildea, R.; Parkhurst, J.; Waterman, D.; Evans, G. Scaling Diffraction Data in the DIALS Software Package: Algorithms and New Approaches for Multi-Crystal Scaling. *Acta Crystallogr. D Struct. Biol.* **2020**, *76* (Pt 4), 385.

(10) Winter, G. Xia2: An Expert System for Macromolecular Crystallography Data Reduction. *J. Appl. Crystallogr.* **2010**, *43* (1), 186–190.

(11) Winter, G.; Waterman, D. G.; Parkhurst, J. M.; Brewster, A. S.; Gildea, R. J.; Gerstel, M.; Fuentes-Montero, L.; Vollmar, M.; Michels-Clark, T.; Young, I. D.; Sauter, N. K.; Evans, G. DIALS: Implementation and Evaluation of a New Integration Package. *Acta Crystallogr. D Struct. Biol.* **2018**, *74* (Pt 2), 85.

(12) McCoy, A. J.; Grosse-Kunstleve, R. W.; Adams, P. D.; Winn, M. D.; Storoni, L. C.; Read, R. J. Phaser Crystallographic Software. *J. Appl. Crystallogr.* **2007**, *40* (Pt 4), 658.

(13) Murshudov, G. N.; Skubák, P.; Lebedev, A. A.; Pannu, N. S.; Steiner, R. A.; Nicholls, R. A.; Winn, M. D.; Long, F.; Vagin, A. A. REFMAC5 for the Refinement of Macromolecular Crystal Structures. *Acta Crystallogr. D Biol. Crystallogr.* **2011**, *67* (Pt 4), 355.

(14) Emsley, P.; Cowtan, K. Coot: Model-Building Tools for Molecular Graphics. *Acta Crystallogr. D Biol. Crystallogr.* **2004**, *60* (Pt 12 Pt 1), 2126–2132.

(15) Agirre, J.; Iglesias-Fernández, J.; Rovira, C.; Davies, G. J.; Wilson, K. S.; Cowtan, K. D. Privateer: Software for the Conformational Validation of Carbohydrate Structures. *Nat. Struct. Mol. Biol.* **2015**, *22* (11), 833–834.

(16) Joosten, R. P.; Long, F.; Murshudov, G. N.; Perrakis, A. The PDB_REDO Server for Macromolecular Structure Model Optimization. *IUCrJ* **2014**, *1* (Pt 4), 213–220.

(17) Schrödinger, L.; DeLano, W. PyMOL. http://www.pymol.org/pymol 2020.

(18) Martyna, G. J.; Klein, M. L.; Tuckerman, M. Nosé–Hoover Chains: The Canonical Ensemble via Continuous Dynamics. *J. Chem. Phys.* **1992**, *97* (4), 2635–2643.

(19) Laskowski, R. A.; Swindells, M. B. LigPlot+: Multiple Ligand-Protein Interaction Diagrams for Drug Discovery. *J. Chem. Inf. Model.* **2011**, *51* (10), 2778–2786.

(20) Jorgensen, W. L.; Chandrasekhar, J.; Madura, J. D.; Impey, R. W.; Klein, M. L. Comparison of Simple Potential Functions for Simulating Liquid Water. *J. Chem. Phys.* **1983**, *79* (2), 926–935.

(21) Jo, S.; Kim, T.; Iyer, V. G.; Im, W. CHARMM-GUI: A Web-Based Graphical User Interface for CHARMM. *J. Comput. Chem.* **2008**, *29* (11), 1859–1865.

(22) Phillips, J. C.; Braun, R.; Wang, W.; Gumbart, J.; Tajkhorshid, E.; Villa, E.; Chipot, C.; Skeel, R. D.; Kalé, L.; Schulten, K. Scalable Molecular Dynamics with NAMD. *J. Comput. Chem.* **2005**, *26*, 1781–1802.

(23) Watowich, S. J.; Meyer, E. S.; Hagstrom, R.; Josephs, R. A Stable, Rapidly Converging Conjugate Gradient Method for Energy Minimization. *J. Comput. Chem.* **1988**, *9* (6), 650–661.

(24) Guvench, O.; Mallajosyula, S. S.; Raman, E. P.; Hatcher, E.; Vanommeslaeghe, K.; Foster, T. J.; Jamison, F. W.; MacKerell, A. D. CHARMM Additive All-Atom Force Field for Carbohydrate Derivatives and Its Utility in Polysaccharide and Carbohydrate-Protein Modeling. *J. Chem. Theory Comput.* **2011**, *7* (10), 3162–3180.

(25) Phillips, J. C.; Hardy, D. J.; Maia, J. D. C.; Stone, J. E.; Ribeiro, J. V.; Bernardi, R. C.; Buch, R.; Fiorin, G.; Hénin, J.; Jiang, W.; McGreevy, R.; Melo, M. C. R.; Radak, B. K.; Skeel, R. D.; Singharoy, A.; Wang, Y.; Roux, B.; Aksimentiev, A.; Luthey-Schulten, Z.; Kalé, L. V.; Schulten, K.; Chipot, C.; Tajkhorshid, E. Scalable Molecular Dynamics on CPU and GPU Architectures with NAMD. *J. Chem. Phys.* **2020**, *153* (4), 44130.

(26) Phillips, J. C.; Zheng, G.; Kumar, S.; Kalé, L. V. NAMD: Biomolecular Simulation on Thousands of Processors. *Proceedings of the International Conference on Supercomputing* **2002**, *2002-November*.

(27) Trott, O.; Olson, A. J. AutoDock Vina: Improving the Speed and Accuracy of Docking with a New Scoring Function, Efficient Optimization and Multithreading. *J. Comput. Chem.* **2010**, *31* (2), 455.

(28) Morris, G. M.; Goodsell, D. S.; Halliday, R. S.; Huey, R.; Hart, W. E.; Belew, R. K.; Olson, A. J. Automated Docking Using a Lamarckian Genetic Algorithm and an Empirical Binding Free Energy Function. *J. Comput. Chem.* **1999**, *19* (14), 16391662.

(29) Reif, M.; Zacharias, M. Computer Modelling and Molecular Dynamics Simulation of Biomolecules. *Biomol. Bioanal. Techn.* **2019**, 501–535.

(30) Blumhagen, K.; Muegge, I.; Knapp, E. W. Diffusion of Two Different Water Models and Thermal Conductivity in a Protein—Water System. *Int. J. Quantum Chem.* **1996**, *59* (4), 271–279.

(31) Feller, S. E.; Zhang, Y.; Pastor, R. W.; Brooks, B. R. Constant Pressure Molecular Dynamics Simulation: The Langevin Piston Method. *J. Chem. Phys.* **1995**, *103* (11), 4613–4621.

(32) Martyna, G. J.; Tobias, D. J.; Klein, M. L.; Martyna, G. J.; Tobias, D. J.; Klein, M. L. Constant Pressure Molecular Dynamics Algorithms. *J. Chem. Phys.* **1994**, *101* (5), 4177–4189.

(33) Mayer, M.; Meyer, B. Characterization of Ligand Binding by Saturation Transfer Difference NMR Spectroscopy. *Angew. Chem. Int. Ed.* **1999**, *38* (12), 1784–1788.

(34) Hwang, T. L.; Shaka, A. J. Water Suppression That Works. Excitation Sculpting Using Arbitrary Wave-Forms and Pulsed-Field Gradients. *J. Magn. Reson. A* **1995**, *112* (2), 275–279. https://doi.org/10.1006/jmra.1995.1047.

(35) Monaco, S.; Tailford, L. E.; Juge, N.; Angulo, J. Differential Epitope Mapping by STD NMR Spectroscopy To Reveal the Nature of Protein–Ligand Contacts. *Angew. Chem. Int. Ed.* **2017**, *56* (48), 15289–15293.

(36) Jubb, H. C.; Higueruelo, A. P.; Ochoa-Montaño, B.; Pitt, W. R.; Ascher, D. B.; Blundell, T. L. Arpeggio: A Web Server for Calculating and Visualising Interatomic Interactions in Protein Structures. *J. Mol. Biol.* **2017**, *429* (3), 365.

(37) Humphrey, W.; Dalke, A.; Schulten, K. VMD: Visual Molecular Dynamics. *J. Mol. Graph.* **1996**, *14* (1), 33–38.
